# *Mitochondrial carrier 1* (*MTCH1*) governs ferroptosis by triggering the FoxO1-GPX4 axis-mediated retrograde signaling in cervical cancer cells

**DOI:** 10.1038/s41419-023-06033-2

**Published:** 2023-08-08

**Authors:** Xuan Wang, Yuting Ji, Jingyi Qi, Shuaishuai Zhou, Sitong Wan, Chang Fan, Zhenglong Gu, Peng An, Yongting Luo, Junjie Luo

**Affiliations:** 1 Department of Nutrition and Health, Beijing Advanced Innovation Center for Food Nutrition and Human Health, China Agricultural University, 100193 Beijing, China; 2 Greater Bay Area Institute of Precision Medicine (Guangzhou), Fudan University, Nansha District, 511400 Guangzhou, China; 3 Institute of Life Sciences, Fudan University, 200433 Shanghai, China

**Keywords:** Cell biology, Cancer

## Abstract

Cervical cancer is one of the leading causes of cancer death in women. Mitochondrial-mediated ferroptosis (MMF) is a recently discovered form of cancer cell death. However, the role and the underlying mechanism of MMF in cervical cancer remain elusive. Here, using an unbiased screening for mitochondrial transmembrane candidates, we identified *mitochondrial carrier 1* (*MTCH1*) as a central mediator of MMF in cervical cancers. *MTCH1*-deficiency disrupted mitochondrial oxidative phosphorylation while elevated mitochondrial reactive oxygen species (ROS) by decreasing NAD^+^ levels. This mitochondrial autonomous event initiated a mitochondria-to-nucleus retrograde signaling involving reduced FoxO1 nuclear translocation and subsequently downregulation of the transcription and activity of a key anti-ferroptosis enzyme glutathione peroxidase 4 (GPX4), thereby elevating ROS and ultimately triggering ferroptosis. Strikingly, targeting *MTCH1* in combination with Sorafenib effectively and synergistically inhibited the growth of cervical cancer in a nude mouse xenograft model by actively inducing ferroptosis. In conclusion, these findings enriched our understanding of the mechanisms of MMF in which *MTCH1* governed ferroptosis though retrograde signaling to FoxO1-GPX4 axis, and provided a potential therapeutic target for treating cervical cancer.

## Introduction

Cervical cancer is one of the most common cancers and the leading cause of cancer death in women, creating an urgent need to develop novel and effective treatment strategies [1]. According to the Global Cancer Statistics 2020, cervical cancer ranks fourth in both incidence (6.5%) and mortality (7.7%) among female cancers, with more than 600,000 new cases and more than 340,000 deaths [2]. Despite emerging therapies have improved cancer survival and reduced recurrence rates, there is still a lack of mature clinical therapeutic targets and strategies for cervical cancer. Therefore, exploring novel effective therapeutic strategies and targets have emerged a particularly critical direction of cervical cancer research.

Ferroptosis is a type of programmed cell death caused by excessive lipid peroxidation [3], and the induction of ferroptosis is considered as a promising anti-cervical cancer therapy mechanism [4, 5]. Mounting studies have found that ferroptosis is closely related to the growth regulation of various cancer cells such as cervical cancer [6], liver cancer [7], ovarian cancer [8], pancreatic cancer [9], and renal cancer [10]. The most common ferroptotic pathway employed in cancer therapy is glutathione peroxidase 4 (GPX4) and glutathione (GSH) signaling [11]. Inhibition of GPX4 with small molecule compounds effectively triggers ferroptosis in tumor cells [12], while exogenous supplementation of GSH shows an opposite effect [13].

Emerging evidence strongly suggests that mitochondria are intimately involved in the regulation of ferroptosis through canonical ferroptosis pathway-independent mechanisms. The mitochondria-associated coenzyme Q10 (CoQ10) oxidoreductase ferroptosis suppressor protein 1 (FSP1) is discovered as a potent ferroptosis-resistance factor acting in parallel to GPX4 in cancer cells [14, 15]. In addition, the dihydroorotate dehydrogenase (DHODH) is another mitochondria-anchored anti-ferroptosis mechanism by reducing ubiquinone to antioxidant ubiquinol independent of GPX4 [16]. Therefore, these mitochondrial-localized defense systems antagonize ferroptosis through a GSH-independent pathway. Given that excessive iron-dependent lipid oxidation is an intrinsic feature of ferroptosis [17], the mitochondrion is a central hub for both oxidative stress and iron homeostasis inside a cell [18, 19], we speculate that mitochondria might be intrinsically and tightly linked to the regulation of ferroptosis through multiple distinct mechanisms. Therefore, exploring the regulatory mechanisms of ferroptosis from a mitochondrial perspective will greatly facilitate the discovery of potential therapeutic targets for cancer treatment.

Mitochondrial transmembrane proteins are critical for mitochondria to maintain normal function, potentially regulating oxidative phosphorylation, oxidative stress and reactive oxygen species (ROS) [20, 21]. In the present study, through an unbiased bioinformatic screening strategy for mitochondrial transmembrane candidates, we identified *mitochondrial carrier 1* (*MTCH1*) as a potential mitochondrial anti-ferroptosis factor in cervical cancers. *MTCH1* deficiency markedly elevated mitochondrial reactive oxygen species (mitoROS) and initiated a mitochondrial retrograde signaling involving the FoxO1-GPX4 axis. We also demonstrated that the combination of *MTCH1* deficiency with the clinical antitumor drug Sorafenib effectively and synergistically induce ferroptosis and suppress cervical cancer growth in a nude mouse xenograft model. This study not only provided a novel perspective on the mechanism of ferroptosis regulation, but also established the groundwork for exploring potential therapeutic targets and strategies for cervical cancers.

## Materials and methods

### Antibodies

The following antibodies were used: MTCH1 (1:1000 dilutions; NBP1-69285, Novus Biologicals, USA); FoxO1 (1:1000 dilutions; 2880, Cell Signaling Technology, USA); p-FoxO1 (1:1000 dilutions; 9464 s, Cell Signaling Technology, USA); Histone (1:1000 dilutions; 17168-1-AP, Proteintech, USA); GAPDH (1:1000 dilutions; 60004-1-Ig, Proteintech, USA); *β*-actin (1:5000 dilutions; 66009-1-Ig, Proteintech, USA); isotype-matched control antibody IgG (1:1000 dilutions; M5409, Sigma-Aldrich, USA). The following secondary antibodies were used: Horseradish peroxide-conjugated goat anti-mouse IgG (H + L) (1:5000 dilutions; A0216, Beyotime Biotechnology, China); goat-anti-rabbit IgG (H + L) (1:5000 dilutions; A0208, Beyotime Biotechnology, China); Alexa Fluor 594 goat anti-rabbit IgG (1:200 dilutions; ab150080, Abcam, UK).

### Reagents

Ferrostatin-1 (347174-05-4), RSL3 (1219810-16-8), Z-VAD (OMe)-FMK (161401-82-7), Necrostatin-1 (4311-88-0) and 3-Methyladenine (5142-23-4) were purchased from TargetMol Chemicals (MA, USA). Propyl iodide (PI, C0080) and fluorescein diacetate (FDA, F8040) were purchased from Solarbio (Beijing, China). BCA Protein Concentration Assay Kit (P0010S) was bought from Beyotime Biotechnology (Jiangsu, China). Mito-TEMPO (1334850-99-5) was bought from Sigma-Aldrich (MO, USA). Plasmid Large Extraction Kit (DC202-01) was purchased from Nanjing Vazyme Biotech (Nanjing, China). FoxO1 activator LOM612 (HY-101035) and inhibitor AS1842856 (HY-100596) was purchased from MedChemExpress (Shanghai, China).

### Bioinformatics analysis and screening

The initial list of 1136 proteins with mitochondrial localization was taken from the MitoCarta3.0 human inventory [22]. Then, 185 multiple transmembrane proteins were selected by predicting the transmembrane structure (*N* ≥ 2) based on TMHMM and topcons-single. For different protein families, the candidate genes form the SLC25 family occupied the largest proportion of multiple transmembrane proteins, hence we further analyzed these genes. Kaplan–Meier survival curve was used to assess the influence of candidate genes from SLC25 family on cervical cancer survival. RFS information was explored in the Kaplan–Meier plotter database (http://kmplot.com/analysis/). The 95% confidence interval hazard ratio and log-rank *P* value were evaluated.

### Cell culture

The human cervical cancer cell line HeLa and Siha cell lines were obtained from the Cell Resource Center of the Institute of Basic Medical Sciences, Chinese Academy of Medical Sciences. HeLa was cultured in Dulbecco’s Modified Eagle’s Medium (11995065, Thermo Fisher Scientific, USA) with high-glucose containing 10% fetal bovine serum (10099141, Thermo Fisher Scientific, USA) while Siha was cultured in Minimum Essential Medium (11095080, Thermo Fisher Scientific, USA) containing 10% fetal bovine serum. Cells were incubated at a constant temperature in a 37 °C cell incubator with 5% CO_2_ and 20% O_2_ in the incubator.

### Gene knockout, gene knockdown and gene overexpression

*MTCH1* gene knockout was performed using CRISPR/Cas9 technology. The synthesized sgRNA (5′-CTCTACTGTGACTCGGGGTA-3′) was linked to the CRISPR-Cas9 plasmid vector, and the plasmid was transfected into HeLa cells by Lipofectamine 3000 Transfection Reagent (L3000015, Thermo Fisher Scientific, USA). 24 h after transfection, GFP positive cells were screened by flow cytometry. The expanded culture clones were sequenced, and frameshift mutations of *MTCH1* indicate successful establishment of gene knockout clones. The lentiviral vectors encoding short hairpin RNAs (shRNAs) targeting *MTCH1* or *FoxO1* were purchased from Hanheng Biotechnology (Shanghai, China), and *MTCH1* or *FoxO1* knock-down clones were established by instructions. *MTCH1* expression plasmid pEnCMV-MTCH1 (human)-Linker-EGFP-SV40-Neo was purchased from MiaoLing Plasmid Sharing Platform (Wuhan, China). *GPX4* expression plasmid was purchased from Sino Biological (Beijing, China). *FoxO1-AAA* expression plasmid was customized from Ji Manchu Biotechnology (Shanghai, China).

### Cell viability assay

Cell viability was measured using the Cell Counting Kit-8 (CCK8) (25126-32-3, TargetMol Chemicals, USA). Cells were inoculated (2000 cells/well) in 96-well plates for 12 h, and then treated with test compounds for indicated time. Cells were washed with PBS, and then medium with 1:9 dilutions of the CCK8 cell titer was stored at 37 °C for 1 h. Absorbance was measured at 450 nm using a microplate reader (Thermo Fisher Scientific, USA).

### PI staining

2 × 10^5^ cells were uniformly seeded into the well plate. The cells were treated without or with specific cell death inhibitors. Then the adherent cells were co-stained with PI and FDA at 37 °C for 20 min. The images were taken under a fluorescence microscope (Zeiss LSM 780, Germany).

### Electron microscopy detection of mitochondrial morphology

Cells (1 × 10^6^) were collected by centrifugation and fixed with 1% osmic acid solution for 1–2 h. Rinse the sample three times with PBS and dehydrated with a gradient concentration of ethanol solutions (30%, 50%, 70%, 80%, 90% and 95%, for 15 min, and then treated with 100% ethanol for 20 min) and acetone treatment for 20 min. The sample was treated with the mixed solution of embedding medium and acetone and heated at 70 °C overnight to obtain embedded samples. The samples were sectioned by Leica EM UC7 ultramicrotome (Leica, Germany), stained with lead citrate solution and 50% uranyl acetate saturated solution in ethanol and dried for transmission electron microscopy in observation.

### Isolation of mitochondria

Mitochondria were isolated using Cell Mitochondria Isolation Kit (C3601, Beyotime Biotechnology, China). In brief, cells were collected and followed by reagent pretreatment: add 1.5 ml mitochondria separation reagent, and then the separated cells were homogenized 30 times. The cells were centrifuged according to manufacturer’s instructions and the sediment is the mitochondria to be harvested.

### ROS detection

ROS was detected by the ROS Assay Kit (S0033S, Beyotime Biotechnology, China). In brief, the DCFH-DA was added to sample suspension according to the concentration of 1:1000 dilutions. Place in 37 °C cell incubator for 20 min. The free DCFH-DA was eliminated by washing with PBS. Detection was performed by fluorescent microplate reader.

### MDA detection

MDA was detected by the MDA Assay Kit (S0131S, Beyotime Biotechnology, China). Briefly, samples were collected and lysed by ultrasonic waves. The mixture was heated in a 100 °C water bath for 60 min, and then the sample was extracted, cooled in an ice bath, and centrifuged for 10 min at a speed of 10,000 *g*. Then the reagents were added, and the absorbance of the supernatant was measured at wavelengths of 450 nm, 532 nm, and 600 nm, respectively. The MDA content was calculated according to the manufacturer’s instructions.

### GSH detection

GSH was detected by GSH and GSSG Detection Kit (S0053, Beyotime Biotechnology, China). In brief, add 1 mL reagent to resuspend samples, repeat freeze-thaw at least 2–3 times, centrifuge at 8000 *g* for 10 min, collect supernatant at 4 °C to be tested. Then the reagents were added, and the absorbance of the supernatant was measured at wavelengths of 412 nm. The GSH content was calculated according to the manufacturer’s instructions.

### NAD^+^ and NADH quantification

NAD^+^ and NADH were quantified by NAD^+^/NADH Detection Kit (S0175, Beyotime Biotechnology, China). In brief, samples were lysed and centrifuged at 12,000 *g* at 4 °C for 10 min, and the supernatant was taken for detection. Then the reagents were added, and the absorbance of the supernatant was measured at wavelengths of 450 nm. NAD^+^ and NADH were calculated according to manufacturer’s instructions.

### ATP quantification

ATP levels were detected using the Enhanced ATP Assay Kit (S0027, Beyotime Biotechnology, China) according to the manufacturer’s protocol. Briefly, the ATP working solution was added to the assay wells at room temperature, followed by the tissue lysis supernatant, and the RLU value was measured with a luminometer after mixing at least 2 s at room temperature.

### Measurement of MMP

MMP was measured using Mitochondria Membrane Potential Detection Kit (JC-10) (CA1310, Beijing Solarbio Science & Technology, China). In brief, add 1 mL of JC-10 staining working solution to the medium, mix well, and incubate in a 37 °C cell incubator for 20 min. After incubation, the supernatant was removed, and the cells were washed twice with JC-10 staining buffer, and add 2 mL medium. Detection was performed by flow cytometry. JC-10 monomer detection: excitation wavelength 490 nm, emission wavelength 530 nm. JC-10 polymer detection: excitation wavelength 525 nm, emission wavelength 590 nm.

### GPX enzyme activity

GPX enzyme activity was detected by the Total Glutathione Peroxidase Assay Kit (S0058, Beyotime Biotechnology, China). Briefly, Cells were collected and lysed with IP cell lysate at a ratio of 100–200 μl of lysate per 1 million cells, followed by centrifugation at 12,000 *g* at 4 °C for 10 min. The supernatant was taken for the determination of enzymatic activity. Using a 96-well plate, add appropriate volume of detection buffer and sample, and incubate at room temperature for 15 min. Add 10 μl of 30 mM peroxide reagent solution to each well and mix well. The absorbance at 340 nm was measured immediately using a microplate reader.

### Real-time quantitative PCR

Trizol reagent (CW0580S, JiangSu CoWin Biotech, China) was used to separate and extract total RNA, and the concentration of RNA was measured by Nanodrop 2000 (Thermo Fisher Scientific, USA). Using HiScript III RT SuperMix (R323-01, Nanjing Vazyme Biotech, China) for reverse transcription, and using Taq Pro Universal SYBR qPCR Master Mix (Q712-02, Nanjing Vazyme Biotech, China) for quantitative PCR in accordance with the manufacturer’s instructions. Real-time quantitative PCR analysis was performed on an Applied Biosystems StepOnePlus real-time PCR instrument (Applied Biosystems, USA). The fold difference in gene expression was calculated using the 2^−ΔΔCt^ method and is presented relative to *ACTB* mRNA. All reactions were performed in triplicate, and specificity was monitored using melting curve analysis. The primer sequences for PCR are summarized in Tab. S1.

### Immunofluorescence and imaging

The cells were cleaned with PBS (each time for 3 min, 3 times) and fixed using 4% paraformaldehyde for 15 min at room temperature. Then the cells were permeabilized by 0.5% Triton X-100 for 10 min at room temperature. The tissues were then blocked with 5% normal goat serum in TBS buffer containing 1% polysorbate 20 (PBST) for 1 h at room temperature. The enclosed liquid was removed then cells were exposed to an antibody for 12 h at 4 °C. On the second day, the coverslips were cleaned by PBST, then exposed to Alexa Fluor 488 secondary antibody for 1 h in Immunohistochemical Wet Box. Finally, DAPI tablets were used to seal the tablets, fluorescence images were viewed by a confocal microscope system (Zeiss LSM 780, Germany), and the results of co-localized pixels were analyzed using LSM version software (Zeiss, Germany).

### Luciferase reporter assay

We constructed the PGL-3 basic plasmid containing *GPX4* promoter region with FoxO1 binding site (pro*-GPX4*) or the sequence with mutation in the binding site (pro-*GPX4*-mut), and co-transfected with empty, *FoxO1* and *FoxO1-AAA* plasmids for fluorescence detection. The FoxO1-AAA is a constitutively active form of FoxO1 [23]. For the luciferase assays, cells at 50% confluency were transfected in 24-well plates with 0.1 μg plasmids. The cells were lysed in 50 mL passive lysis buffer after transfection for 48 h. The soluble fraction was subsequently assayed for luciferase activity with a Dual-Luciferase Reporter Assay System (E2940, Promega Corporation, USA) following the manufacturers’ protocols.

### Chromatin immunoprecipitation (ChIP) analysis

Specific protein-DNA interactions were detected by ChIP followed by qPCR (Chromatin Immunoprecipitation Assay Kit, 17-295, Sigma-Aldrich, USA). 2 × 10^6^ cells were uniformly seeded into the well plate, and the target protein and corresponding genomic DNA were cross-linked by fixation with 1% formaldehyde at room temperature for 10 min. The extracted DNA-protein complex was sheared by the sonicator to a length of between 200 and 500 base pairs. The resulting fragment was incubated with 1 µg FoxO1-specific antibody or IgG (as a negative control) in slow oscillations overnight, then immunoprecipitated with protein A + G. Specific reagents were used to wash the precipitation, and then Protease K was added and incubated at 45 °C for 1 h. The DNA was then recovered with QIAquick PCR purification kit (28104, QIAGEN, Germany) for qPCR to demonstrate affinity for the *GPX4* promoter region. Quantitative PCR was performed for the *GPX4* promoter with the potential FoxO1 binding site. The primers of predicted FoxO1 binding site were 5′-GCCTGTTGTCCCAGCTACTC-3′ and 5′-GGGGTTTGGTTTCTCCAACT-3′. The primers of *GAPDH* as a control were 5′-CGGAGTCAACGGATTTGGTCGTAT-3′ and 5′-AGCCTTCTCCATGGTGGTGAAGAC-3′.

### Protein extraction and immunoblot

Cells were washed with ice cold-PBS and cleaved directly in a 2 × Lemmli sample buffer containing DTT (78442, Thermo Fisher Scientific, USA) or in a RIPA buffer containing a stop protease and phosphatase inhibitor (9806, Cell Signaling Technology, USA). The protein samples were separated by 10% sodium dodecyl sulfate-polyacrylamide gel electrophoresis and transferred to 0.45 μm PVDF membrane. Seal the membrane with 5% BSA or 5% buttermilk in Tris buffered saline containing 0.1% twen-20 for 1 h. Specific primary antibodies were incubated overnight at 4 °C, and then tested with horseradish peroxisase-coupled anti-mouse or anti-rabbit secondary antibodies. All western blots were performed using a chemiluminescent reagent (Thermo pierce, USA) and signals were collected by ChemiScope3600MINI (Clinx Scientific Instrument, China).

### Xenograft mouse models

HeLa cells were selected for routine trypsin digestion, and the cell concentration was adjusted to 2 × 10^7^/ml. Six-week-old male BALB/c nude mice (Beijing Vital River Laboratory Animal Technology, China) were randomly selected and 0.3 ml of cell suspension was injected subcutaneously under the left flank. Sorafenib (T0093L, TargetMol Chemicals, USA) and a control saline were administered after 2 weeks of tumor bearing. And Solafenib was injected intraperitoneally for 14 days (10 mg/kg/day). The body weight and tumor size of nude mice were monitored. The tumor volume was measured by an electronic caliper using the formula as follows: tumor volume (mm^3^) = length (mm) × width (mm) × height (mm) × π/6. After 14 days of Solafenib administration, mice were euthanized and the tumor tissues were taken for pathological observation and analysis. All test procedures were abided by the Guiding Principles for the Care and Use of Laboratory Animals and reviewed and approved by the Committee on the Ethics of Animal Experiments of China Agricultural University (Beijing, China; Approval Code: AW81103202-4-1).

### Statistical analysis

Data presented were expressed as mean ± standard error of mean (SEM) of three or more biological replicates/biologically independent experiments. All statistical calculations were analyzed according GraphPad Prism 8 software. The difference between two conditions was compared using an unpaired Student’s *t* test, and one-way analysis of variance (ANOVA) was used to compare three or more conditions. Significant difference: *P < 0.05, **P < 0.01, ***P < 0.001.

## Results

### *MTCH1* was a potent ferroptosis suppressor in cervical cancer

**Fig. 1:**
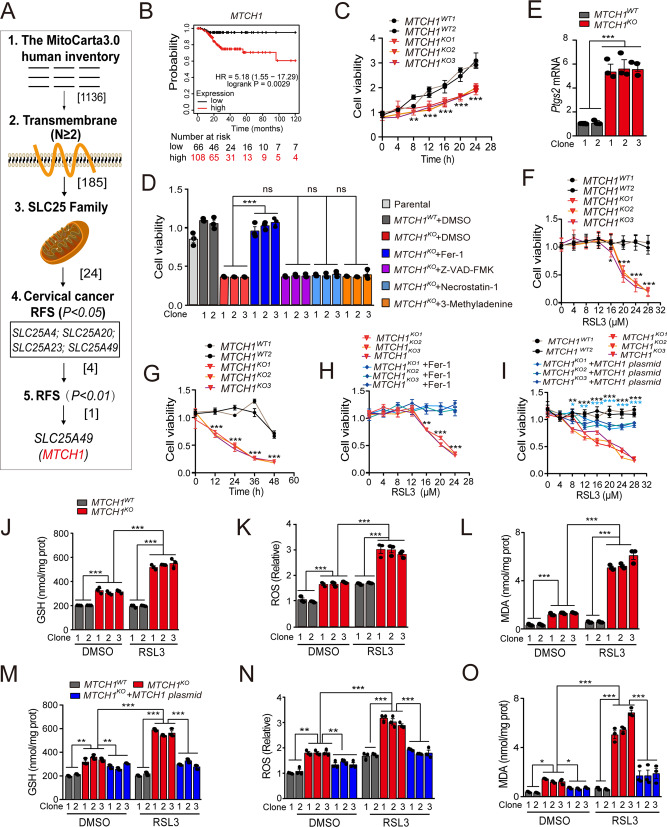
An unbiased bioinformatic screen identified *MTCH1* as a ferroptosis-resistance factor in cervical cancers. **A** Summary chart of the workflow for the identification of *MTCH1*. **B** Analysis of the association between the *MTCH1* transcriptional level and RFS in cervical cancer from the Kaplan–Meier plotter database. **C** Relative cell viability of *MTCH1*^*WT*^ (*n* = 2) and *MTCH1*^*KO*^ (*n* = 3) HeLa clones at different times. **D** Relative cell viability of *MTCH1*^*WT*^ (*n* = 2) and *MTCH1*^*KO*^ (*n* = 3) HeLa clones treated with different cell death inhibitors for 24 h. **E** Transcriptional level of *Ptgs2* in *MTCH1*^*WT*^ (*n* = 2) and *MTCH1*^*KO*^ (*n* = 3) HeLa clones. **F** Relative cell viability of *MTCH1*^*WT*^ (*n* = 2) and *MTCH1*^*KO*^ (*n* = 3) HeLa clones treated with different concentrations (0, 4, 8, 12, 16, 20, 24 and 28 µM) of RSL3 for 24 h. **G** Relative cell viability of *MTCH1*^*WT*^ (*n* = 2) and *MTCH1*^*KO*^ (*n* = 3) HeLa clones treated with 20 µM RSL3 for different times (0, 12, 24, 36 and 48 h). **H** Relative cell viability of *MTCH1*^*KO*^ (*n* = 3) HeLa clones treated with different concentrations of RSL3 with or without 10 µM Fer-1 for 24 h. **I** Relative cell viability of *MTCH1*^*WT*^ (*n* = 2), *MTCH1*^*KO*^ (*n* = 3) and *MTCH1*^*KO*^ transfected with *MTCH1* plasmid (*n* = 3) HeLa clones treated with different concentrations of RSL3 for 24 h. * in black means the compare of *MTCH1*^*WT*^ and *MTCH1*^*KO*^, and * in cyan means the compare of *MTCH1*^*KO*^ and *MTCH1*^*KO*^ transfected with *MTCH1* plasmid. **J–L** Relative levels of GSH (**J**), ROS (**K**), and mitochondrial MDA (**L**) in *MTCH1*^*WT*^ (*n* = 2) and *MTCH1*^*KO*^ (*n* = 3) HeLa clones treated with RSL3 (20 µM) or not for 24 h. **M–O** Relative levels of GSH (**M**), ROS (**N**), and mitochondrial MDA (**O**) in *MTCH1*^*WT*^ (*n* = 2), *MTCH1*^*KO*^ (*n* = 3) and *MTCH1*^*KO*^ transfected with *MTCH1* plasmid (*n* = 3) HeLa clones treated with RSL3 (20 µM) or not for 24 h. Data were presented as mean ± SEM of at least three independent replicates (*P < 0.05, ** *P* < 0.01, *** *P* < 0.001) and analyzed by one-way ANOVA with Tukey’s multiple comparisons test or unpaired *t*-test.

To figure out the potential mitochondrial candidates that are associated with cervical cancer, a bioinformatic analysis and screening strategy was designed (Fig. 1A). An inventory of 1136 candidates with mitochondrial localization was obtained by MitoCarta3.0 [22], and 185 proteins with at least secondary transmembrane (*N* ≥ 2) were obtained by predicting the transmembrane structure of protein. Further analysis revealed that the SLC25 family members (24) constitute the majority of 185 candidates. Analysis of these SLC25 proteins in the Kaplan–Meier plotter database [24] showed that the transcriptional levels of four candidate genes were correlated with relapse-free survival (RFS) of cervical cancer (*P* < 0.05), with *MTCH1* had the most particularly pronounced effect (*P* < 0.01) (Fig. 1A). In addition, cervical cancer patients with low transcription of *MTCH1* had a higher probability of RFS (HR = 5.18 (1.55–17.29), *P* = 0.0029) (Fig. 1B), suggesting that low expression of *MTCH1* possibly benefited cervical cancer patients and improved the prognosis.

To explore whether *MTCH1* might play an inhibitory role in cervical cancer, we generated *MTCH1* knockout (*MTCH1*^*KO*^) and *MTCH1* knockdown (*MTCH1*^*KD*^) cervical cancer cell lines in HeLa or Siha (Fig. S1A–D; Supplementary File; Tab. S2). The cell viability was significantly decreased upon *MTCH1* downregulation (*P* < 0.01) (Fig. 1C; Fig. S1E). To clarify which type of cell death is associated with reduced cell viability, rescue experiments were performed using the ferroptosis inhibitor Ferrostatin-1 (Fer-1) [25], apoptosis inhibitor Z-VAD-FMK [26], necrosis inhibitor Necrostatin-1 [27] and autophagy inhibitor 3-Methyladenine [28] (Fig. 1D). Only Fer-1 completely rescued cell death while other inhibitors had no effects (Fig. 1D). In addition, PI staining further confirmed that ferroptosis is associated with reduced cell viability upon *MTCH1* downregulation (Fig. S1E; Fig. S2A). Furthermore, *MTCH1*-deficient cells had higher levels of the ferroptosis-related gene *prostaglandin-endoperoxide synthase 2* (*Ptgs2*) [29] (Fig. 1E), suggesting that the reduced cell viability in *MTCH1*-deficient cells might be attributed to ferroptosis. Cell viability experiments further revealed a profound increase in the sensitivity of *MTCH1*-deficient cells to the ferroptosis inducer RSL3 [30] in a dose- and time-dependent manner, which was reduced by Fer-1 supplement or expression of *MTCH1* (Fig. 1F–I). The content of GSH and ROS was significantly increased in *MTCH1*^*KO*^ and *MTCH1*^*KD*^ cervical cancer cells (Fig. 1J–K; Fig. S2B–D). Concomitantly, the levels of malondialdehyde (MDA) were significantly increased in mitochondria but not the whole cells of *MTCH1*^*KO*^ and *MTCH1*^*KD*^ clones (Fig. 1L; Fig. S2E,F). Furthermore, overexpression of *MTCH1* in *MTCH1*^*KO*^ clones restored the contents of GSH and ROS in whole cells and MDA in mitochondria (Fig. 1M–O; Fig. S1G,H). Collectively, these results suggested that *MTCH1* was a potent ferroptosis suppressor in cervical cancers.

### *MTCH1*-deficiency induced ferroptosis by inhibiting GPX4 expression and activity in cervical cancer cells

**Fig. 2:**
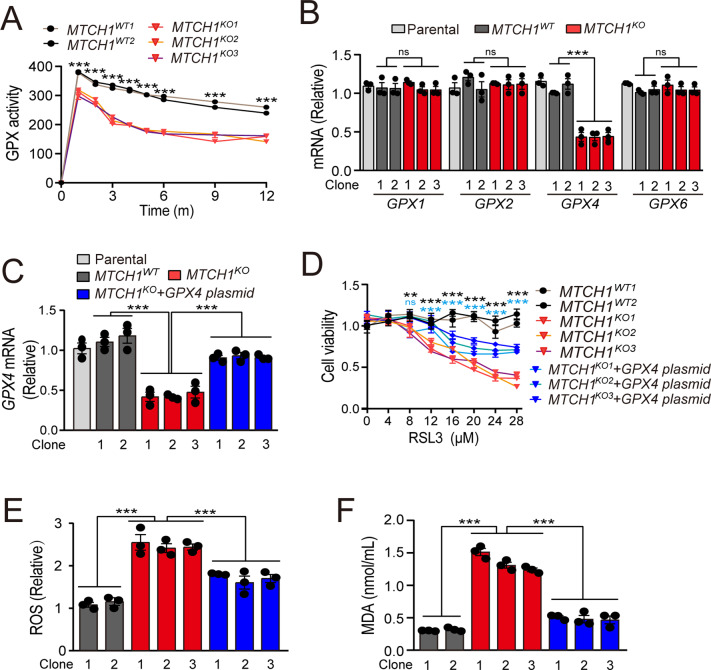
*MTCH1*-deficiency induced ferroptosis of cervical cancer cells by inhibiting GPX4 activity. **A** GPX activity in *MTCH1*^*WT*^ (*n* = 2) and *MTCH1*^*KO*^ (*n* = 3) HeLa clones. **B** Relative transcriptional levels of *GPX1*, *GPX2*, *GPX4* and *GPX6* in Parental (*n* = 1), *MTCH1*^*WT*^ (*n* = 2), and *MTCH1*^*KO*^ (*n* = 3) HeLa clones. **C** Relative transcriptional levels of *GPX4* in Parental (*n* = 1), *MTCH1*^*WT*^ (*n* = 2), *MTCH1*^*KO*^ (*n* = 3) and *MTCH1*^*KO*^ transfected with *GPX4* plasmid (*n* = 3) HeLa clones. **D** Relative cell viability in *MTCH1*^*WT*^ (*n* = 2), *MTCH1*^*KO*^ (*n* = 3) and *MTCH1*^*KO*^ transfected with *GPX4* plasmid (*n* = 3) HeLa clones treated with different concentrations of RSL3 (0, 4, 8, 12, 16, 20, 24, 28 µM) for 24 h. * in black means the compare of *MTCH1*^*WT*^ and *MTCH1*^*KO*^ clones, and * in cyan means the compare of *MTCH1*^*KO*^ and *MTCH1*^*KO*^ clones transfected with *GPX4* plasmid. **E**,**F** Relative levels of ROS (**E**) and mitochondrial MDA (**F**) in *MTCH1*^*WT*^ (*n* = 2), *MTCH1*^*KO*^ (*n* = 3) and *MTCH1*^*KO*^ transfected with *GPX4* plasmid (*n* = 3) HeLa clones. Data were presented as mean ± SEM of at least three independent replicates (**P < 0.01, ***P < 0.001, ns, no significant) and analyzed by one-way ANOVA with Tukey’s multiple comparisons test or unpaired *t*-test.

We next explored the molecular basis for enhanced ferroptosis upon *MTCH1*-deficiency. Since GPX family members play a pivotal role in ferroptosis by detoxifying lipid peroxides [31], we then measured the activity and expression of GPX. GPX activity measurements showed that *MTCH1*^*KO*^ clones had lower GPX activity (Fig. 2A). Consistently, the transcriptional levels of GPX4 were dramatically reduced in *MTCH1*^*KO*^ clones of both HeLa and Siha, while the expression of *GPX1*, *GPX2* and *GPX6* remained unchanged (Fig. 2B; Fig. S2I). Moreover, overexpression of GPX4 restored resistance to RSL3 (Fig. 2C,D), with concomitant decrease of both ROS and mitochondrial MDA in *MTCH1*-deficient clones (Fig. 2E,F). These results indicated that *MTCH1*-deficiency induced ferroptosis probably through GPX4 inhibition in cervical cancer cells.

### *MTCH1*-deficiency-induced ferroptosis was associated with impaired mitochondrial OXPHOS and mitoROS in cervical cancer cells

**Fig. 3:**
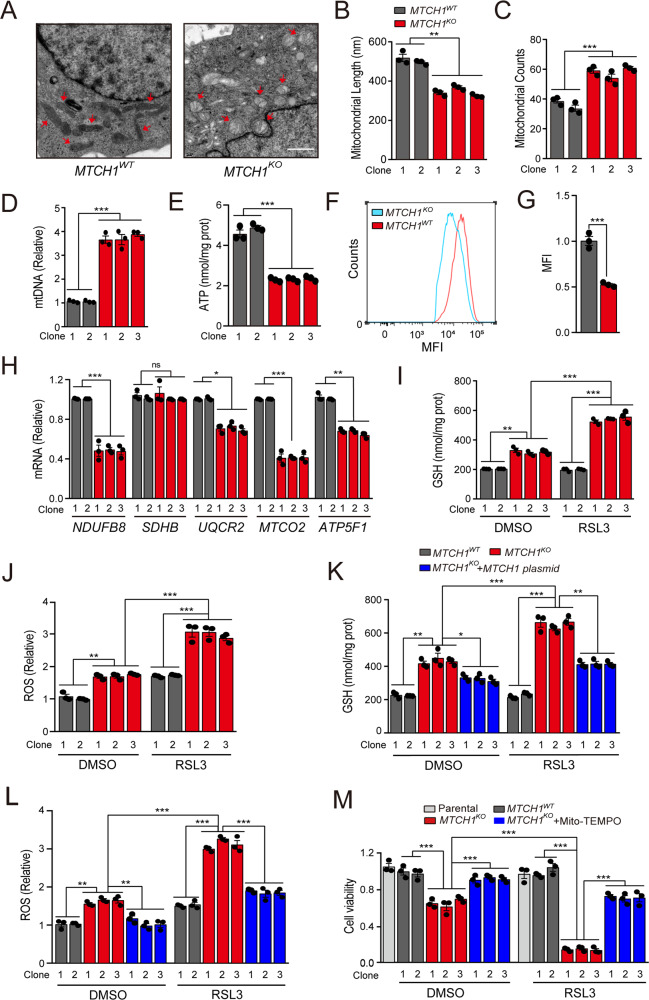
*MTCH1*-deficiency induced ferroptosis was linked to mitochondria damages. **A** Representative TEM imaging of the mitochondria in *MTCH1*^*WT*^ and *MTCH1*^*KO*^ HeLa clones. Scale bar, 1 μm. Red arrows indicate mitochondria. **B**, **C** Mitochondrial length (**B**) and mitochondrial counts (**C**) in *MTCH1*^*WT*^ (*n* = 2) and *MTCH1*^*KO*^ (*n* = 3) HeLa clones. **D** Relative levels of mitochondria DNA copy number (mtDNA) in *MTCH1*^*WT*^ (*n* = 2) and *MTCH1*^*KO*^ (*n* = 3) HeLa clones. **E** ATP levels in *MTCH1*^*WT*^ (*n* = 2) and *MTCH1*^*KO*^ (*n* = 3) HeLa clones. **F**, **G** Mitochondrial membrane potential (MMP) (**F**) and corresponding statistical analysis (**G**) in *MTCH1*^*WT*^ and *MTCH1*^*KO*^ HeLa clones. **H** Relative transcriptional levels of *NDUFB8*, *SDHB*, *UQCR2*, *MTCO2* and *ATP5F1* in *MTCH1*^*WT*^ (*n* = 2) and *MTCH1*^*KO*^ (*n* = 3) HeLa clones. **I**, **J** Levels of mitochondrial GSH (**I**) and ROS (**J**) in *MTCH1*^*WT*^ (*n* = 2) and *MTCH1*^*KO*^ (*n* = 3) HeLa clones treated with RSL3 (20 µM) or DMSO for 24 h. **K**, **L** Relative levels of mitochondrial GSH (**K**) and ROS (**L**) in *MTCH1*^*WT*^ (*n* = 2), *MTCH1*^*KO*^ (*n* = 3) and *MTCH1*^*KO*^ transfected with *MTCH1* plasmid (*n* = 3) HeLa clones treated with RSL3 (20 µM) or DMSO for 24 h. **M** Relative cell viability in Parental (*n* = 1), *MTCH1*^*WT*^ (*n* = 2), *MTCH1*^*KO*^ (*n* = 3) and *MTCH1*^*KO*^ added with Mito-TEMPO (*n* = 3) HeLa clones treated with RSL3 (20 µM) or DMSO for 24 h. Data were presented as mean ± SEM of at least three independent replicates (*P < 0.05, **P < 0.01, ***P < 0.001, ns, no significant) and analyzed by one-way ANOVA with Tukey’s multiple comparisons test or unpaired *t*-test.

*MTCH1* has been reported as mitochondrial outer membrane protein and might control mitochondrial biogenesis [32]. We hypothesized that *MTCH1*-deficiency would have negative effects on mitochondria. As expected, electron microscopy (EM) analysis showed abnormal mitochondrial morphology with a swollen spherical shape and internal vacuoles in *MTCH1*^*KO*^ cells compared with *MTCH1*^*WT*^ cells (Fig. 3A,B). Although both mitochondrial number (Fig. 3C) and the copy number of mitochondrial DNA (mtDNA) (Fig. 3D) were elevated, we observed a reduction of adenosine triphosphate (ATP) production (Fig. 3E; Fig. S3A) and mitochondrial membrane potential (MMP) (Fig. 3F,G), with concomitant downregulation of mitochondrial respiratory chain complex related genes (*NDUFB8*, *UQCR2*, *MTCO2* and *ATP5F1*) (Fig. 3H) in *MTCH1*^*KO*^ cells. These results supported a structural and functional defect of mitochondria upon *MTCH1* inactivation.

We next explored whether *MTCH1*-deficiency-induced ferroptosis was associated with mitochondrial dysfunction. We found elevated levels of both mitochondrial GSH and mitoROS in *MTCH1*^*KO*^ cells (Fig. 3I,J). This increase could be restored by overexpression of MTCH1 (Fig. 3K,L). In addition, the reduced cell viability in *MTCH1*^*KO*^ clones was efficiently restored by mitochondrial antioxidant Mito-TEMPOL [33] in the presence or absence of RSL3 (Fig. 3M). These results suggested a possible linkage between *MTCH1*-deficiency-induced ferroptosis and mitochondrial dysfunction.

### *MTCH1*-deficiency led to increased mitoROS and decreased GPX4 expression through downregulating mitochondrial NAD^+^ levels

**Fig. 4:**
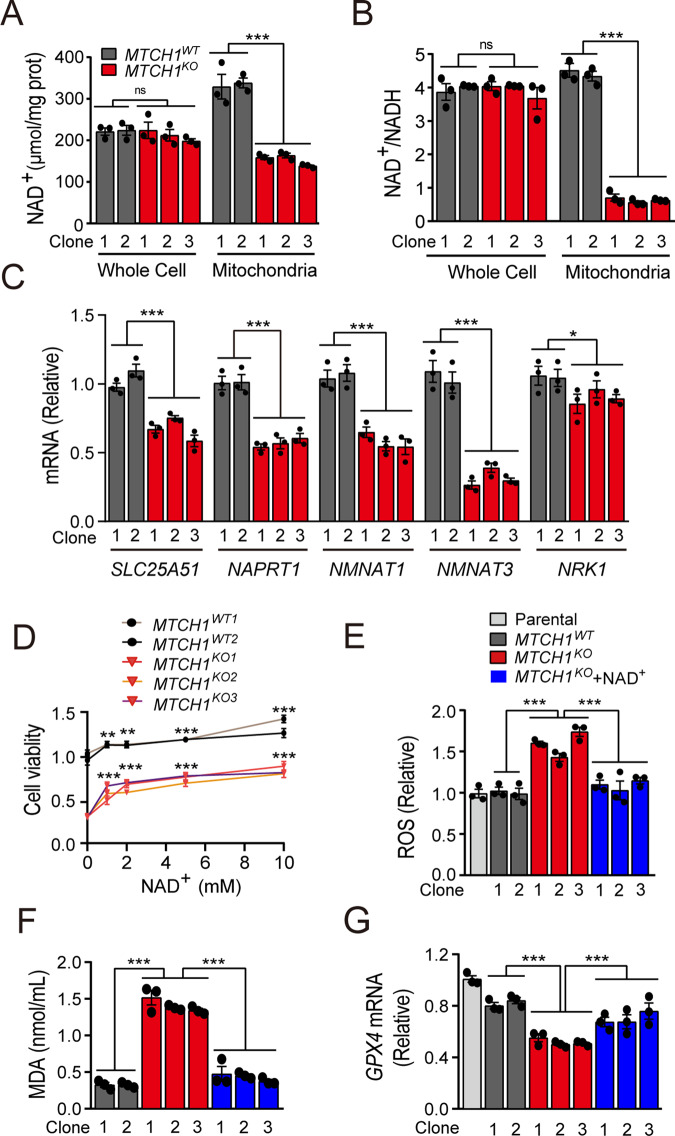
*MTCH1*-deficiency reduced mitochondrial NAD^+^ levels, leading to ROS elevation and GPX4 inhibition. **A**, **B** NAD^+^ (**A**) and NAD^+^/NADH (**B**) levels in whole cells or mitochondria lysates of *MTCH1*^*WT*^ (*n* = 2) and *MTCH1*^*KO*^ (*n* = 3) HeLa clones. **C** Relative transcriptional levels of *SLC25A51*, *NMNAT1*, *NMNAT3*, *NAPRT1* and *NRK1* in *MTCH1*^*WT*^ (*n* = 2), *MTCH1*^*KO*^ (*n* = 3) HeLa clones. **D** Relative cell viability in *MTCH1*^*WT*^ (*n* = 2) and *MTCH1*^*KO*^ (*n* = 3) HeLa clones treated with different concentrations of NAD^+^ (0, 1, 2, 5, 10 mM) for 24 h. * refers to the compare of NAD^+^ treated and untreated clones. **E**, **F** The levels of ROS (**E**) and mitochondrial MDA (**F**) in *MTCH1*^*WT*^ (*n* = 2), *MTCH1*^*KO*^ (*n* = 3), and *MTCH1*^*KO*^ treated with 5 mM NAD^+^ for 24 h (*n* = 3) HeLa clones. **G** Relative transcriptional levels of *GPX4* in Parental (*n* = 1), *MTCH1*^*WT*^ (*n* = 2), *MTCH1*^*KO*^ (*n* = 3), and *MTCH1*^*KO*^ treated with 5 mM NAD^+^ for 24 h (*n* = 3) HeLa clones. Data were presented as mean ± SEM of at least 3 independent replicates (**P < 0.01, ***P < 0.001, ns, no significant) and analyzed by one-way ANOVA with Tukey’s multiple comparisons test or unpaired *t*-test.

NAD^+^ levels are closely related to mitochondrial function [34, 35]. Since *MTCH1*-deficiency resulted in mitochondrial dysfunction, we next measured NAD^+^ and NAD^+^/NADH content at both cellular and mitochondrial levels in *MTCH1*-deficient HeLa and Siha clones. The results showed a remarkable decrease in both NAD^+^ and NAD^+^/NADH levels in mitochondria but not whole cell of *MTCH1*^*KO*^ and *MTCH1*^*KD*^ clones (Fig. 4A,B; Fig. S3B–E). To figure out how MTCH1 regulates NAD^+^ level, we measured the transcriptional levels of NAD^+^ synthesis related genes (*NMNAT1*, *NMNAT3*, *NAPRT1*, *NRK1*, *NNMT* and *NAMPT*) and NAD^+^ transporter gene *SLC25A51* [36, 37]. We found that the transcriptional levels of *SLC25A51*, *NMNAT1*, *NMNAT3*, *NAPRT1* and *NRK1* in *MTCH1*^*KO*^ HeLa clones were significantly down-regulated (Fig. 4C; Fig. S3F,G), suggesting that MTCH1 regulates mitochondrial NAD^+^ levels by possibly influencing NAD^+^ synthesis and transport. In addition, supplementation with NAD^+^ restored cell viability (Fig. 4D) and attenuated ferroptosis in *MTCH1*^*KO*^ HeLa clones as manifested by reduced mitoROS and mitochondrial MDA as well as elevated GPX4 expression (Fig. 4E–G). Thus, mitochondrial NAD^+^ was necessary and sufficient to confer ferroptosis resistance.

### *MTCH1*-deficiency initiated pro-ferroptotic retrograde signaling involving the FoxO1-GPX4 axis in cervical cancer cells

**Fig. 5:**
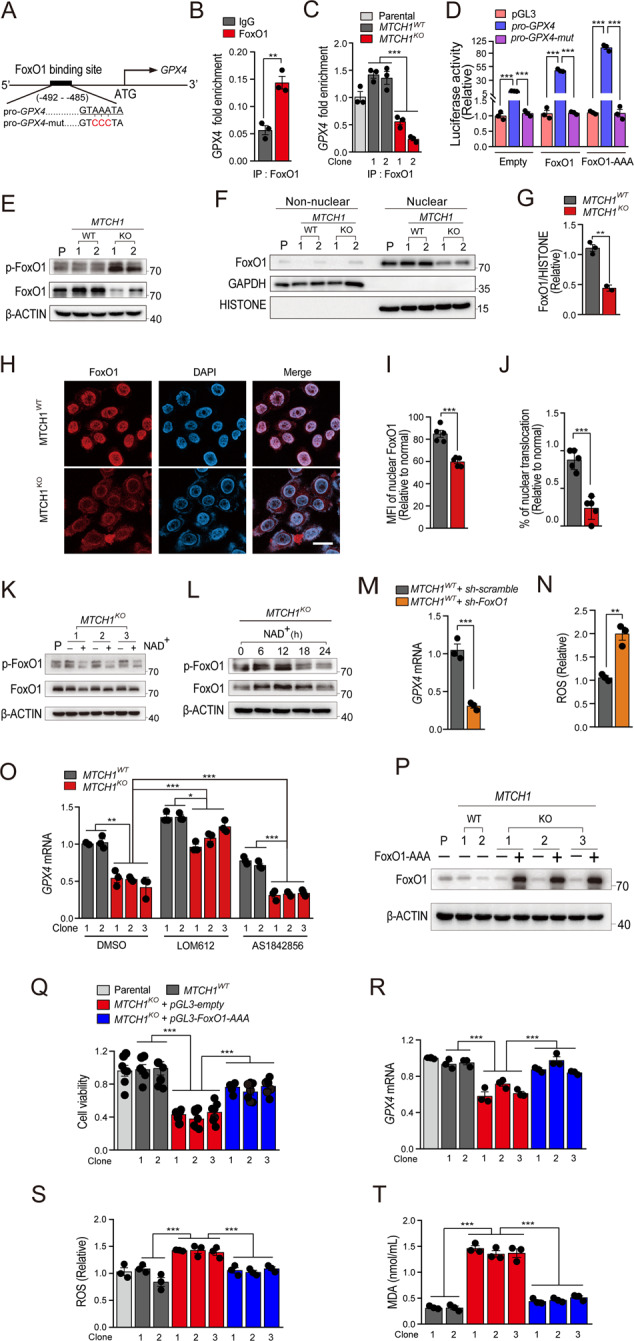
Decreased NAD^+^ levels activated the FoxO1 phosphorylation to inhibit *GPX4* expression and promote ferroptosis. **A** Design of luciferase reporter vector containing human *GPX4* promoter with a FoxO1 binding site (*pro-GPX4*) or a mutant FoxO1 binding site (pro-GPX4-mut). The red indicates mutant bases. **B** ChIP assay using IgG or FoxO1 antibody and quantification of the enrichment of FoxO1 binding to *GPX4* promoter in HeLa cells. **C** ChIP assay using FoxO1 antibody and quantification of the enrichment of FoxO1 binding to *GPX4* promoter in Parental (*n* = 1), *MTCH1*^*WT*^ (*n* = 2), and *MTCH1*^*KO*^ (*n* = 2) HeLa clones. **D** Luciferase reporter assay of FoxO1 binding to *GPX4*. The FoxO1 binding site was shown in (**A**). **E** Immunoblots of lysates from Parental (*n* = 1), *MTCH1*^*WT*^ (*n* = 2), and *MTCH1*^*KO*^ (*n* = 2) HeLa clones with the indicated antibodies. **F** Immunoblots of lysates from the nuclear and cytoplasmic (non-nuclear) of Parental (*n* = 1), *MTCH1*^*WT*^ (*n* = 2), and *MTCH1*^*KO*^ (*n* = 2) HeLa cell clones with the indicated antibodies. **G** Quantitative analysis of FoxO1 in the nucleus (**F**) relative to Histone. **H** Representative fluorescence imaging (*n* = 6 images in each group) of FoxO1 (red) and nucleus (blue) in *MTCH1*^*WT*^ and *MTCH1*^*KO*^ HeLa clones. Scale bar, 100 µm. **I**, **J** The mean fluorescence intensity (MFI) of nuclear FoxO1 (**I**) and the nuclear translocation percentage of FoxO1 (**J**) in *MTCH1*^*WT*^ and *MTCH1*^*KO*^ HeLa clones. **K** Immunoblots of lysates from Parental (*n* = 1) and *MTCH1*^*KO*^ (*n* = 3) HeLa clones treated with or without 5 mM NAD^+^ with the indicated antibodies. **L** Immunoblots of lysates from *MTCH1*^*KO*^ HeLa clones treated with 5 mM NAD^+^ for different time (0, 6, 12, 18, 24 h) with the indicated antibodies. **M**, **N** Transcriptional levels of *GPX4* (**M**) and ROS content (**N**) in HeLa cells transfected with *sh-scramble* or *sh-FoxO1* plasmid. **O** Transcriptional levels of *GPX4* in *MTCH1*^*WT*^ (*n* = 2) and *MTCH1*^*KO*^ (*n* = 3) HeLa clones treated with DMSO, FoxO1 activator LOM612 or FoxO1 inhibitor AS1842856. **P** Immunoblots of lysates from Parental (*n* = 1), *MTCH1*^*WT*^ (*n* = 2), and *MTCH1*^*KO*^ (*n* = 3) HeLa clones transfected with FoxO1-AAA plasmid or not with indicated antibodies. **Q–T** Relative cell viability (**Q**), *GPX4* transcriptional levels (**R**), ROS content (**S**) and mitochondrial MDA content (**T**) in Parental (*n* = 1), *MTCH1*^*WT*^ (*n* = 2), and *MTCH1*^*KO*^ transfected with FoxO1-AAA plasmid or empty vector (*n* = 3) HeLa clones. Data were presented as mean ± SEM of at least three independent replicates (*P < 0.05, **P < 0.01, ***P < 0.001) and analyzed by one-way ANOVA with Tukey’s multiple comparisons test or unpaired *t*-test.

The results above suggested that *MTCH1* inactivation reduced mitochondrial NAD^+^, thereby promoted ferroptosis through downregulating *GPX4* transcription. We then explored regulatory mechanism in *GPX4* transcription upon *MTCH1*-deficiency. Examination of human *GPX4* gene revealed a potential FoxO1 binding site (GTAAATA) (Fig. 5A) located 485 bp upstream of *GPX4* transcription start site. ChIP assays showed an enrichment of the *GPX4* DNA fragment by anti-FoxO1 antibody in *MTCH1*^*WT*^ HeLa cells (Fig. 5B), whereas this enrichment was dramatically reduced in *MTCH1*^*KO*^ clones (Fig. 5C; Fig. S4A), suggesting the requirement of *MTCH1* in the binding of FoxO1 to *GPX4* promoter. To confirm this binding, we cloned a 1 kb fragment of the 5′ region of *GPX4* containing this binding site into the luciferase reporter vector pGL3. HeLa cells transfected with the *GPX4* reporter vector demonstrated significantly increased luciferase activity after FoxO1 and FoxO1-AAA (constitutively active FoxO1) overexpression, whereas the luciferase activity of cells transfected with the mutant *GPX4* vector remained at basal levels (Fig. 5D).

We next explored whether *MTCH1* is required for FoxO1 nuclear translocation and activation. The phosphorylation of FoxO1 at Thr24 residue inhibits its nuclear translocation [38]. We observed higher p-FoxO1 with a concomitant lower FoxO1 expression in both nuclear and whole cell lysates in *MTCH1*^*KO*^ clones (Fig. 5E–J; Fig. S4B–D; Supplementary File). Moreover, supplementation with NAD^+^ restored p-FoxO1 in *MTCH1*^*KO*^ cells (Fig. 5K,L; Supplementary File), suggesting a requirement of *MTCH1* and NAD^+^ for FoxO1 activation.

To further confirm the role of FoxO1 in *GPX4* expression and ferroptosis, we performed a series of experiments. Firstly, knockdown of FoxO1 in *MTCH1*^*WT*^ HeLa cells reduced *GPX4* transcription and increased ROS production (Fig. 5M,N). Secondly, FoxO1 activator LOM612 [39] restored *GPX4* levels in *MTCH1*^*KO*^ clones, while inhibitor AS1842856 [40] showed an opposite effect (Fig. 5O). Consistently, overexpression of constitutively active FoxO1-AAA restored cell viability and *GPX4* expression, and reduced ROS and mitochondrial MDA levels (Fig. 5P–T; Supplementary File).

These results suggested that *MTCH1*-deficiency reduced mitochondrial NAD^+^ levels and inhibited FoxO1 activation, which subsequently downregulated *GPX4* transcription and accumulated ROS, ultimately triggering ferroptosis in cervical cancer cells.

### Targeting *MTCH1* synergistically promotes the antitumor activity of Sorafenib

**Fig. 6:**
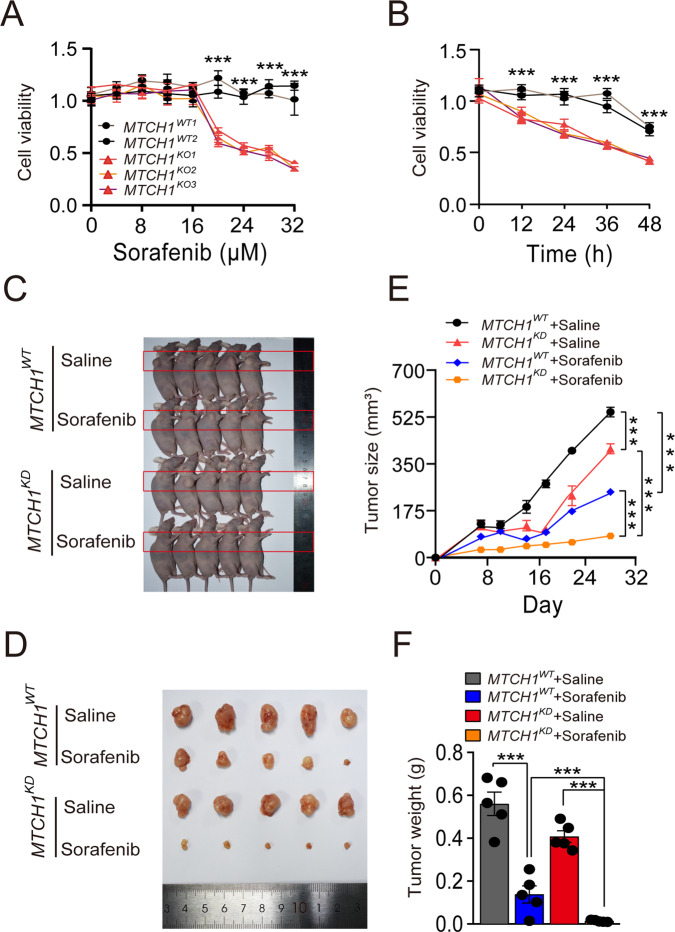
Targeting *MTCH1* promoted the antitumor activity of Sorafenib. **A** Relative cell viability of *MTCH1*^*WT*^ (*n* = 2) and *MTCH1*^*KO*^ (*n* = 3) HeLa clones treated with different concentrations (0, 4, 8, 12, 16, 20, 24, 28, 32 µM) of Sorafenib for 24 h. **B** Relative cell viability of *MTCH1*^*WT*^ (*n* = 2) and *MTCH1*^*KO*^ (*n* = 3) HeLa clones treated with 20 µM Sorafenib for different time (0, 12, 24, 36, 48 h). **C** Representative image of nude mice with tumor from different *MTCH1*^*WT*^ and *MTCH1*^*KD*^ HeLa cells implantation or treatment groups (*n* = 5). **D** Representative image of tumors from different *MTCH1*^*WT*^ and *MTCH1*^*KD*^ HeLa cells implantation or treatment groups of nude mice (*n* = 5). **E** The tumor volume of different *MTCH1*^*WT*^ and *MTCH1*^*KD*^ HeLa cells implantation or treatment groups of nude mice with time (*n* = 5). **F** The tumor weight of different *MTCH1*^*WT*^ and *MTCH1*^*KD*^ HeLa cells implantation or treatment groups of nude mice (*n* = 5). Data were presented as mean ± SEM of at least 3 independent replicates (***P < 0.001) and analyzed by one-way ANOVA with Tukey’s multiple comparisons test or unpaired *t*-test.

Given that Sorafenib is a multi-target drug and ferroptosis inducible antitumor agent [41, 42], and that *MTCH1* inactivation triggers ferroptosis, we investigated whether targeting *MTCH1* in combination with Sorafenib could produce an additive effect on inhibiting tumor growth. We found that Sorafenib reduced cell viability in *MTCH1*-deficiency cells in a dose- and time-dependent manner (Fig. 6A,B). We then developed a nude mouse xenograft model using *MTCH*^*WT*^ and *MTCH1*^*KD*^ HeLa cells. Sorafenib treatment was started when tumors reached a diameter of 5–6 mm. Our results showed that the growth of human cervical cancers was markedly suppressed by *MTCH1* deficiency, Sorafenib treatment and *MTCH1* deficiency + Sorafenib treatment (Fig. 6C). A significant reduction in the tumor volume and weight was observed in the *MTCH1* deficiency or Sorafenib-treated group compared with the control group (Fig. 6D–F), suggesting a role for *MTCH1* in cervical cancer growth. It is interesting that the combinatorial targeting of *MTCH1* and Sorafenib treatment showed a significant inhibitory effect on the growth of cervical cancer (97.8%) that was more efficient than *MTCH1*-deficiency (27.3%) or Sorafenib (75.6%) alone (Fig. 6D–F; *P* < 0.001, combined treatment versus *MTCH1*-deficiency; *P* < 0.001, combined treatment versus Sorafenib), suggesting that the *MTCH1*-deficiency and Sorafenib acted, at least in part, on different pathways. Besides, there was no significant difference in the body weight between each group, indicating that *MTCH1*-deficiency and Sorafenib addition did not harm the growth of nude mice while inhibit cervical cancer (Fig. S5). In conclusion, our results suggested that *MTCH1*-deficiency impaired cervical cancer growth in vivo, and this effect was more pronounced when combined with Sorafenib.

## Discussion

**Fig. 7:**
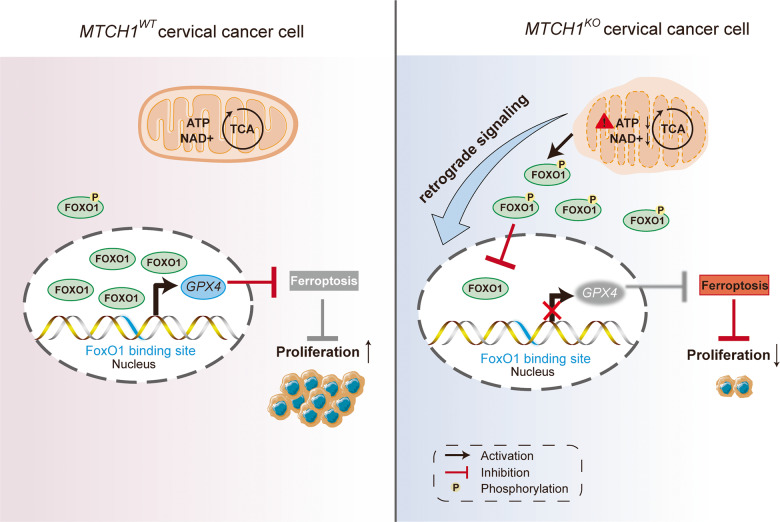
Schematic illustration of *MTCH1* in cervical cancers via governing ferriptosis by retrograde signaling involving the FoxO1-GPX4 axis. (*Left*) When *MTCH1* was expressed normally (*MTCH1*^*WT*^), the mitochondria maintained normal function and numerous FoxO1 transcription factors entered the nucleus to promote *GPX4* transcription, followed by inhibition of ferroptosis. As a result, the proliferation of cervical cancer cells was not inhibited. (*Right*) When *MTCH1* expression was defective (*MTCH1*^*KO*^), the mitochondrial function was abnormal, including mitochondrial morphological and structural changes, decreased levels of ATP and NAD^+^, resulting in significant phosphorylation of FoxO1 transcription factors and inability to enter the nucleus, thereby inhibiting *GPX4* transcription. As a result, ferroptosis was triggered and the proliferation of cervical cancer cells was reduced.

Most of the current reports of *MTCH1* focus on its role as an important regulator of apoptosis [43–45], leaving additional biological activities unexplained. Notably, a recent study shows that up-regulation of *MTCH1* expression is associated with the proliferation, invasion and migration of liver cancer cells [46], suggesting that *MTCH1* may have other potential new functions in cancer. In this study, we provided four noteworthy contributions. Firstly, we expanded the biological functions of *MTCH1* and delineated a novel molecular mechanism by which *MTCH1* negatively regulated ferroptosis in cervical cancer cells. Mechanistically, *MTCH1*-deficiency impaired mitochondrial dysfunction and decreased NAD^+^ levels, which subsequently reduced FoxO1 nuclear translocation, thereby inhibiting the expression and activity of GPX4, resulting in abnormal accumulation of ROS and ultimately ferroptosis (Fig. 7). Secondly, the concept of FoxO1 as a transcription factor of *GPX4* and the FoxO1-GPX4 regulatory axis was first proposed and demonstrated. Thirdly, different from the GPX4-independent NADH-FSP1-CoQ10 [14] and DHODH [16] pathways, we identified MTCH1-FoxO1-GPX4 as a novel mitochondrial retrograde signaling pathway to govern ferroptosis. This is a new link between mitochondria and ferroptosis, which has great scientific implications for understanding how mitochondria regulate ferroptosis. Fourthly, targeting *MTCH1* in combination with Sorafenib effectively and synergistically inhibited the growth of cervical cancer. Altogether, these observations indicated that *MTCH1* might serve as an attractive therapeutic target for cervical cancer.

By bioinformatics analysis and screening, we identified *MTCH1* from a large number of mitochondrial localization proteins as being most closely associated with RFS of cervical cancer. In fact, *MTCH1* (*SLC25A49*) is a member of the SLC25 family with a vital role in transporting nutrients across the inner mitochondrial membrane for energy conversion and cell maintenance [47]. Another member *SLC25A51* from the same family has been shown to be a transporter of NAD^+^/NADH [37], and it is speculated that it may be related to the development of cancer by regulating cellular metabolism. Intriguingly, *SLC25A11* and *SLC25A29* have also been reported to be involved in cancer progression by affecting mitochondrial function [48, 49]. Programmed cell death is believed to play a crucial role in the development and treatment of various cancers. Previous reports have focused on the relationship between *MTCH1* and apoptosis, however, little has been reported about the SLC25 family and ferroptosis. Here, we provided strong evidence to link *MTCH1* with ferroptosis and cancer growth, and explored potential mechanisms and clinical applications.

Maintaining the mitochondrial NAD^+^ pool and an optimal NAD^+^/NADH ratio are essential for mitochondrial homeostasis and proper cellular function [50, 51]. We observed that in the absence of MTCH1, mitochondrial NAD^+^ concentration and NAD^+^/NADH ratio were in decline, mitochondrial morphology and number were changed, MMP was decreased, ATP production was reduced, and ROS level was raised. When supplemented with NAD^+^ in *MTCH1*-deficient cells, a significant reduction in ROS was found. Consistent with our results, previous reports indicate that NAD^+^ emerges as an important regulator in maintaining cellular ROS levels, and ROS accumulates when NAD^+^ is depleted [52, 53]. Furthermore, rescue of *MTCH1* expression restored both the NAD^+^ and ATP content in mitochondria, with a concomitant reduction of ROS levels. Therefore, *MTCH1-*deficiency led to elevated ROS production, presumably due to NAD^+^ depletion in mitochondria.

The transcriptional factor FoxO1 has been implicated in a wide range of biological processes [54]. It is phosphorylated by Akt and translocated from the nucleus to the cytoplasm, where it stays and degrades to lose its transcriptional activity [55]. FoxO1 is thought to be closely related to the apoptosis and autophagy pathway [54, 56, 57]. However, its role in ferroptosis remains largely unknown. Here, we provided the first evidence that FoxO1 was involved in the regulation of ferroptosis by acting as a transcriptional factor to regulate the expression and activity of the key ferroptosis-related enzyme GPX4. Unfortunately, although we observed that *MTCH1*-deficiency-induced reduction of NAD^+^ level activated the FoxO1 phosphorylation and reduced FoxO1 nuclear translocation, how *MTCH1* regulates NAD^+^ level will require future investigation.

GSH and GPX4 are essential for the control of ferroptosis [30]. GSH serves as an important substrate for antioxidant GPX4, which prevents the accumulation of lipid ROS [58]. In general, GSH is insufficient during the process of ferroptosis [59]. However, our results indicated that it was elevated during ferroptosis induced by *MTCH1*-deficiency, especially in combination with ferroptosis inducer RSL3 treatment. This was possibly attributed to different regulatory mechanisms of ferroptosis. In our study, the accumulation of GSH may be due to the inhibition of GPX4 expression and activity, while GSH as the substrate cannot be fully utilized and consumed.

The growth and proliferation rate of cervical cancer cells with *MTCH1* gene knockout was extremely low, and we found that they could not form tumors when transplanted into nude mice, suggesting that *MTCH1* is critical for cervical cancer development. Thus, we compromisingly utilized the *MTCH1* knockdown cell line to establish a nude mouse tumor-bearing model. Sorafenib, an FDA-approved multikinase inhibitor drug, can kill a variety of cancer cells (such as liver, pancreas, colon and kidney cancers) by inducing ferroptosis. Recently, a study reports that Sorafenib can also be used to inhibit cervical cancer cells [6]. In order to explore more effective clinical treatment strategies and harness ferroptosis for therapeutic benefit, we targeted *MTCH1* in combination with Sorafenib to fight against cervical cancer. Our results suggested that this novel strategy can synergistically inhibit the growth of cancer cells and have an excellent anti-cervical cancer therapeutic effect both at cellular level and in animals, providing valuable potential applications.

Taken together, these previously unappreciated mechanistic insights that *MTCH1* governs ferroptosis in cervical cancer open up new avenues for the development of anti-cancer drugs and strategies.

## Supplementary information

Supplementary material

Supplemental File Original Blots

Reproducibility checklist

Author change confirmation

## Data Availability

The data that support the findings of this study are available in this paper and the supplementary material.
