# Supplementary material for: Mitochondrial carrier 1 (MTCH1) governs ferroptosis by triggering the FoxO1-GPX4 axis-mediated retrograde signaling in cervical cancer cells
Source: Cell Death Dis. 2023 Aug 8;14(8):508. doi: 10.1038/s41419-023-06033-2 (PMC10406804; doi:10.1038/s41419-023-06033-2)
Supplement: Supplementary file 1 — Supplementary material [file 41419_2023_6033_MOESM1_ESM.docx]

**Supplementary material**

**Figure S1**


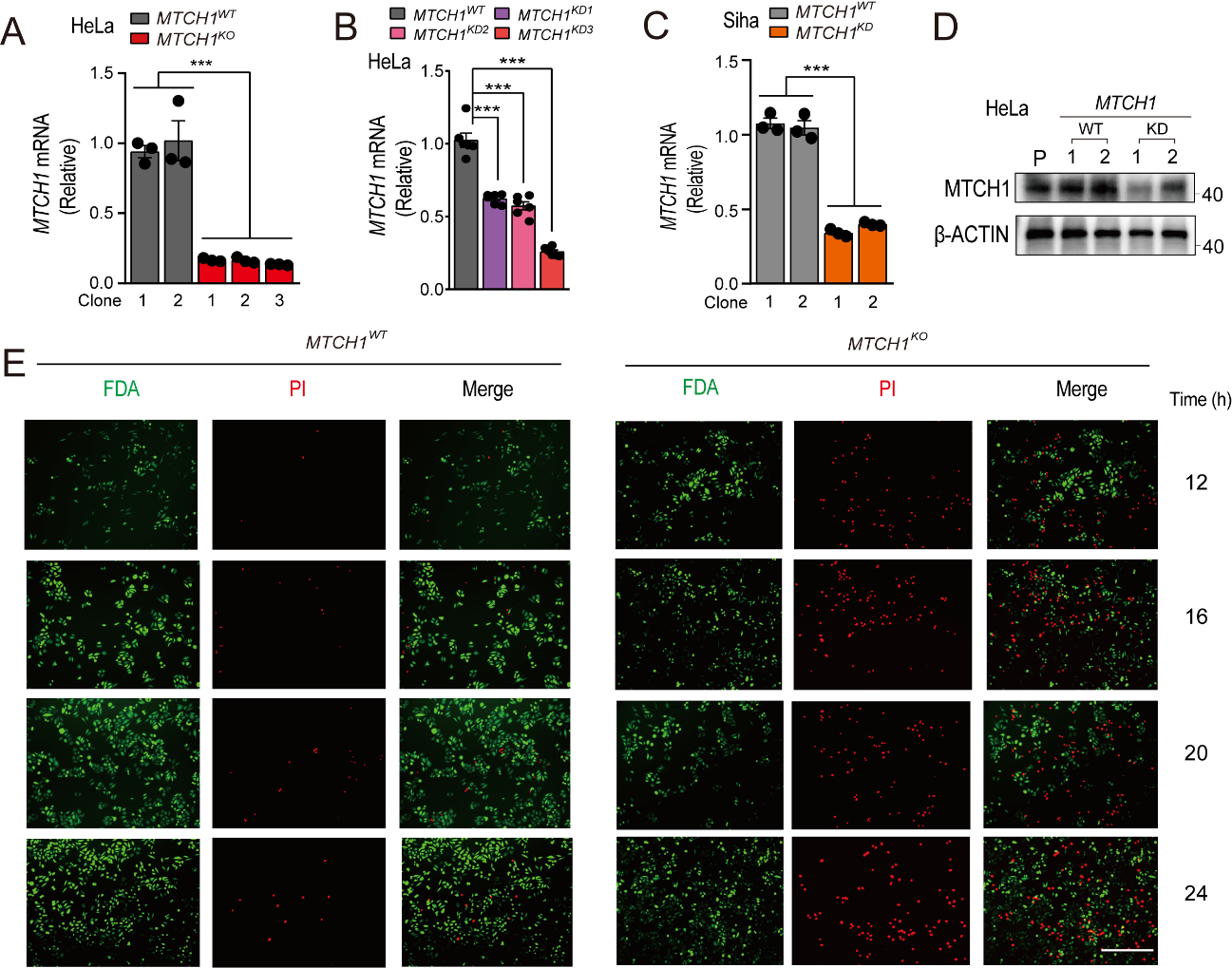


**Figure S1.** Construction of *MTCH1*-deficiency cervical cancer cell lines. (**A**) Relative transcriptional levels of *MTCH1* in *MTCH1^WT^* (n = 2) and *MTCH1^KO^* (n = 3) HeLa clones. (**B**) Relative transcriptional levels of *MTCH1* in *MTCH1^WT^* (n = 1) and *MTCH1^KD^* (n = 3) HeLa clones. (**C**) Relative transcriptional levels of *MTCH1* in *MTCH1^WT^* (n = 2) and *MTCH1^KD^* (n = 2) Siha clones. (**D**) Immunoblots of Parental (n = 1), *MTCH1^WT^* (n = 2) and *MTCH1^KD^* (n = 2) HeLa clones with the MTCH1 antibody. (**E**) PI staining of *MTCH1^WT^* and *MTCH1^KO^* HeLa clones at different time points after cell plating (n = 3). Data are presented as mean ± SEM of at least 3 independent replicates (*** *P* < 0.001; ns, no significant) and analyzed by one-way ANOVA with Tukey’s multiple comparisons test or unpaired t test.

**Figure S2**


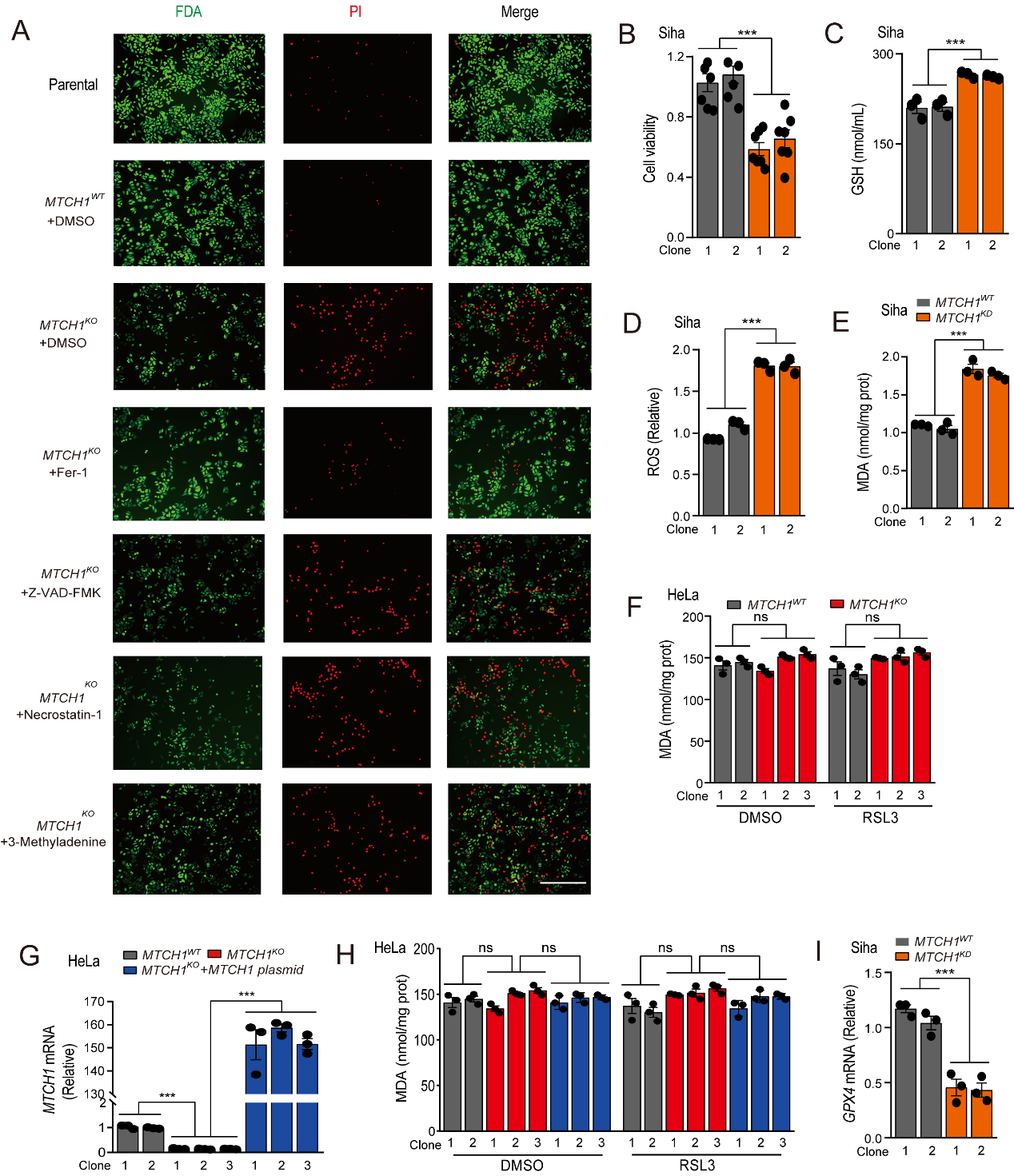


**Figure S2.** Detection of ferroptosis related indexes. (**A**) PI staining of parental cell, *MTCH1^WT^* and *MTCH1^KO^* HeLa clones treated without or with different cell death inhibitors for 24 hours (n = 3). (**B**-**D**) Relative cell viability (B), GSH content (C) and ROS levels (D) in *MTCH1^WT^* (n = 2) and *MTCH1^KD^* (n = 2) Siha clones. (**E**) Mitochondrial MDA content in *MTCH1^WT^* (n = 2) and *MTCH1^KD^* (n = 2) Siha clones. (**F**) Levels of MDA in *MTCH1^WT^* (n = 2) and *MTCH1^KO^* (n = 3) HeLa clones treated with DMSO or 20 µM RSL3 for 24 hours. (**G**) Relative transcriptional levels of *MTCH1* in *MTCH1^WT^* (n = 2), *MTCH1^KO^* (n = 3) and *MTCH1^KO^* transfected with *MTCH1* plasmid (n = 3) HeLa clones. (**H**) MDA content in *MTCH1^WT^* (n = 2), *MTCH1^KO^* (n = 3) and *MTCH1^KO^* transfected with *MTCH1* plasmid (n = 3) HeLa clones treated with DMSO or 20 µM RSL3 for 24 hours. (**I**) Relative transcriptional levels of *GPX4* in *MTCH1^WT^* (n = 2) and *MTCH1^KD^* (n = 2) Siha clones. Data are presented as mean ± SEM of 3 independent replicates (*** *P* < 0.001) and analyzed by one-way ANOVA with Tukey’s multiple comparisons test.

**Figure S3**


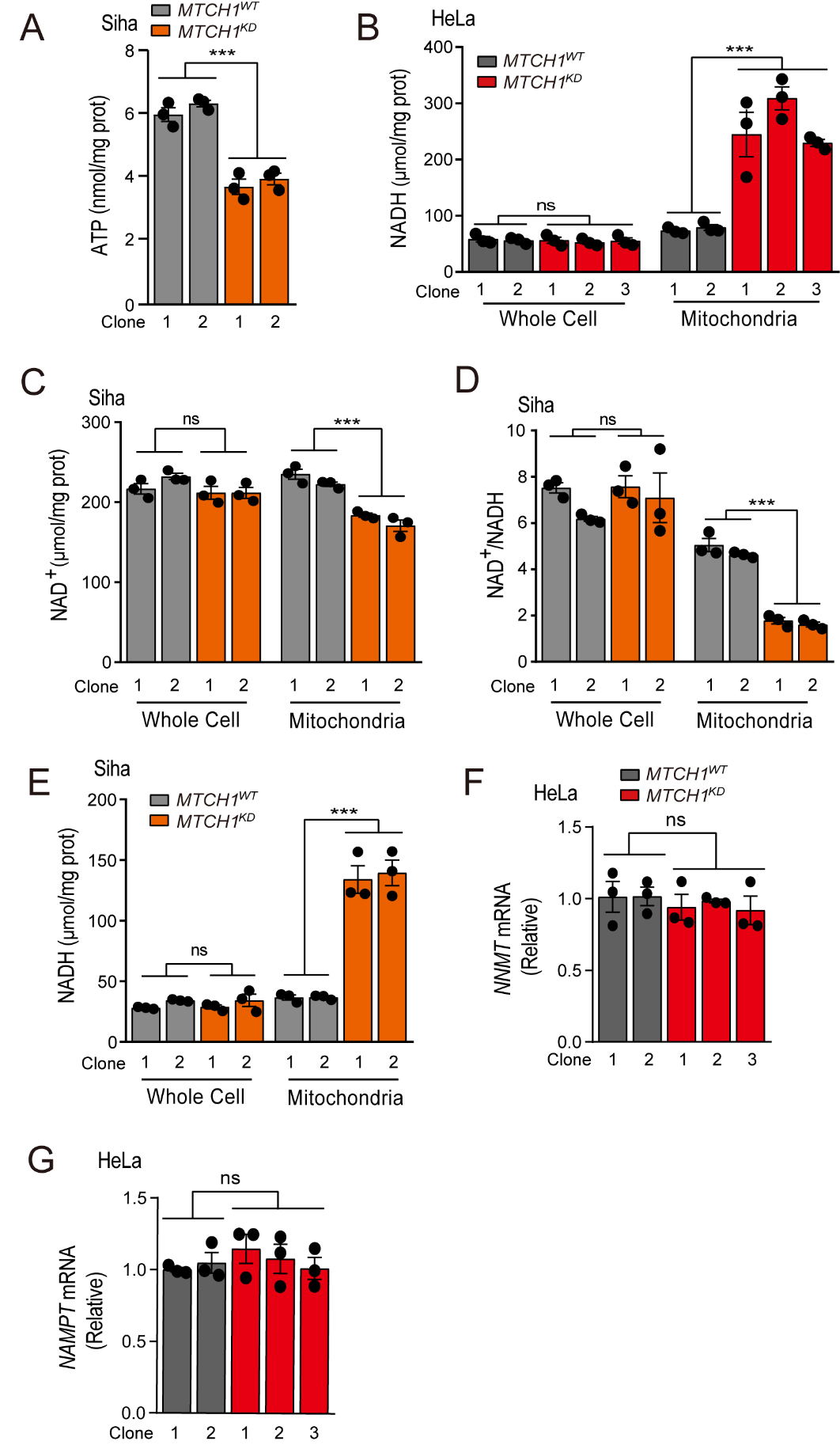


**Figure S3.** *MTCH1*-deficiency impairs mitochondrial function. (**A**) ATP levels in *MTCH1^WT^* (n = 2) and *MTCH1^KD^* (n = 2) Siha clones. (**B**) NADH content in whole cell or mitochondria lysates of *MTCH1^WT^* (n = 2) and *MTCH1^KO^* (n = 3) HeLa clones. (**C-E**) NAD^+^ (C), NAD^+^/NADH (D) and NADH (E) content in whole cell or mitochondria lysates of *MTCH1^WT^* (n = 2) and *MTCH1^KD^* (n = 2) Siha clones. (**F**-**G**) Relative transcriptional levels of *NNMT* (F) and *NAMPT* (G) in *MTCH1^WT^* (n = 2), *MTCH1^KO^* (n = 3) HeLa clones. Data are presented as mean ± SEM of at least 3 independent replicates (****P* < 0.001; ns, no significant) and analyzed by one-way ANOVA with Tukey’s multiple comparisons test or unpaired t test.

**Figure S4**

**
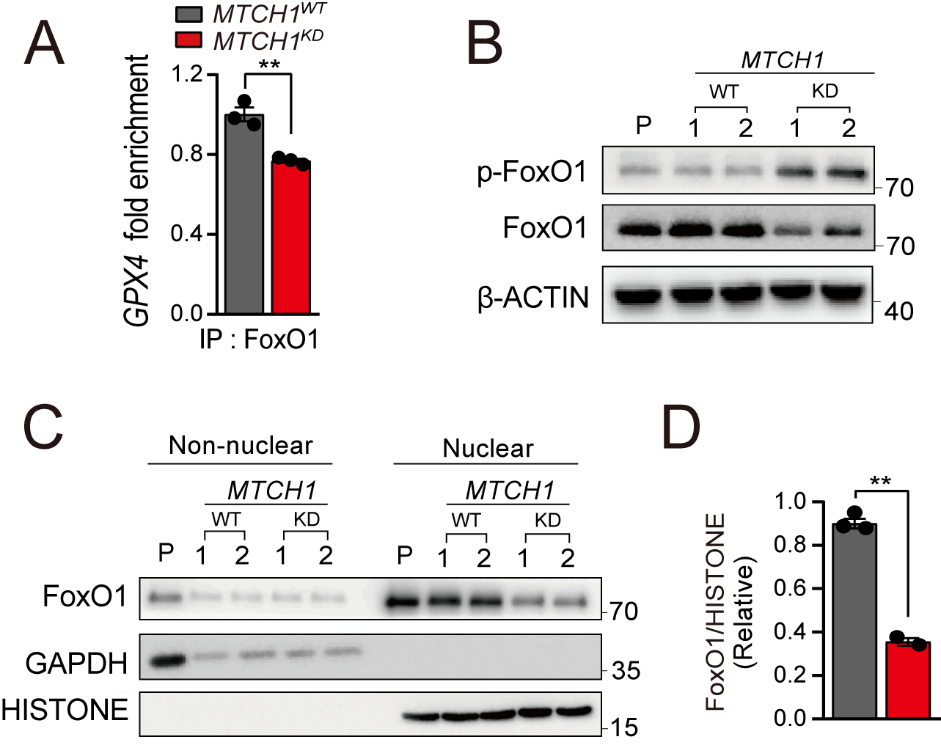
**

**Figure S4.** *MTCH1*-deficiency regulates FoxO1 activity in HeLa cells. (**A**) ChIP assay using FoxO1 antibody and quantification of the enrichment of FoxO1 binding to *GPX4* promoter in *MTCH1^WT^* and *MTCH1^KD^* HeLa clones. (**B**) Immunoblots of lysates from Parental (n = 1), *MTCH1^WT^* (n = 2), and *MTCH1^KD^* (n = 2) HeLa clones with the indicated antibodies. (**C**) Immunoblots of lysates from the nuclear and cytoplasmic (non-nuclear) of Parental (n = 1), *MTCH1^WT^* (n = 2), and *MTCH1^KD^* (n = 2) HeLa cell clones with the indicated antibodies. (**D**) Quantitative analysis of FoxO1 in the nucleus in (C) relative to Histone. Data are presented as mean ± SEM of at least 3 independent replicates (***P* < 0.01) and analyzed by one-way ANOVA.

**Figure S5**


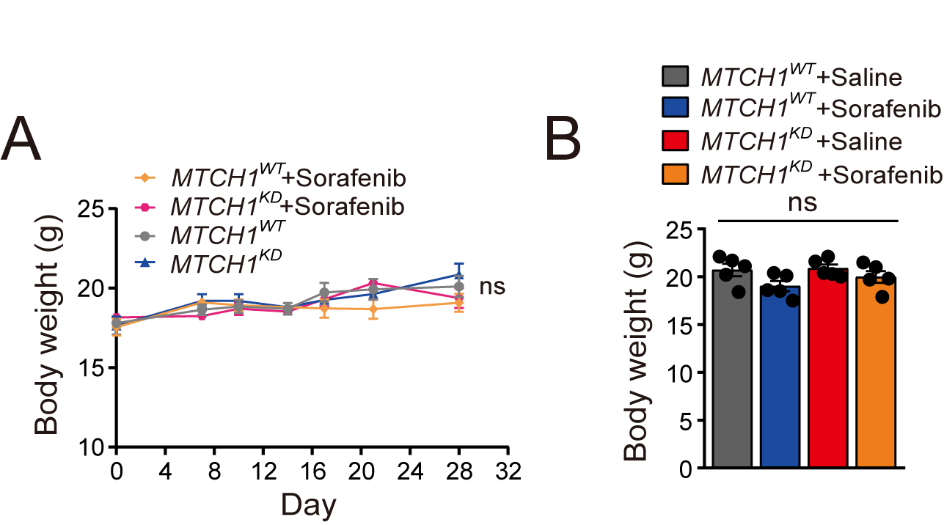


**Figure S5.** The body weight of different *MTCH1^WT^* and *MTCH1^KD^* HeLa cells implantation or treatment groups of nude mice with time (**A**) or before execution (**B**) (n = 5). ns, no significant. Data are analyzed by one-way ANOVA with Tukey’s multiple comparisons test or unpaired t test.

**Table S1.** Primers for quantitative PCR.

| Gene | Forward / Reverse sequence |
| --- | --- |
| *ACTB*  *MTCH1* | CTCACCATGGATGATGATATCGC / AGGAATCCTTCTGACCCATGCC  TGAGGCAGAGAGGTATCAGGA / GATTCCCAGAGGTCACGTTCC |
| *Ptgs2* | GTTCCACCCGCAGTACAGAA / AGGGCTTCAGCATAAAGCGT |
| *GPX1* | TATCGAGAATGTGGCGTCCC / TCTTGGCGTTCTCCTGATGC |
| *GPX2* | TGAATGGGCAGAACGAGCAT / TCCGGCCCTATGAGGAACTT |
| *GPX4* | AGATCCAACCCAAGGGCAAG / GACGGTGTCCAAACTTGGTG |
| *GPX6* | TGCTCAGCAGACCCTAAAGC / AATAGGCGGCCACATTGACA |
| *hB2M* | TGCTGTCTCCATGTTTGATGTATCT / TCTCTGCTCCCCACCTCTAAGT |
| *mtDNA* | CACCCAAGAACAGGGTTTGT / TGGCCATGGGTATGTTGTTA |
| *NDUFB8* | GTTGAACTGGGGTGAACCGA / GAGAAGTGGTGCCTACACACA |
| *SDHB* | GTGGCCCCATGGTATTGGAT / CGGGTGCAAGCTAGAGTGTT |
| *UQCR2* | CCTGCGGGGTGATGTTGATA / CAGCTACTTCCCAACGACGA |
| *ATP5F1* | TCACAGGGACGCTAAGATTGC / AGGCTGCATTCTTCAGAGAGG |
| *MTCO2* | CCGTCTGAACTATCCTGCCC / GAGGGATCGTTGACCTCGTC |
| *SLC25A51* | CGCTGATGGGAAATCCAGTTA / CTGGAGTTTGGCAGGATGATAG |
| *NMNAT1* | AAGCTGTGCCAAAGGTCAAG / TTCCAGCCCGAGTAACACAT |
| *NMNAT3* | GGATGGAGACAGTGAAGGTGCT / GTCGAGAAGAGTGCCTTGCCAT |
| *NAPRT1* | AGCCACGAATGAAGCTGACCGA / CACTGGCTCTTCTGCTAACTGC |
| *NRK1* | CCAAATTGCAGTGTCATATCTCAG / CCAGCAGGAAATGGCTGACATC |
| *NNMT* | GTTTGGTTCTAGGCACTCTGCAG / AGAGCCGATGTCAATCAGCAGG |
| *NAMPT* | AGGGTTACAAGTTGCTGCCACC / CTCCACCAGAACCGAAGGCAAT |

**Table S2.** *MTCH1* gene mutations in HeLa clones.

| Clone | Allele 1 | Allele 2 |
| --- | --- | --- |
| KO1 | -1 bp | +5 bp |
| KO2 | -1 bp | -1 bp |
| KO3 | -1 bp | -1 bp |
