# Supplementary material for: Mitochondrial carrier 1 (MTCH1) governs ferroptosis by triggering the FoxO1-GPX4 axis-mediated retrograde signaling in cervical cancer cells
Source: Cell Death Dis. 2023 Aug 8;14(8):508. doi: 10.1038/s41419-023-06033-2 (PMC10406804; doi:10.1038/s41419-023-06033-2)
Supplement: Supplementary file 2 — Supplemental File Original Blots [file 41419_2023_6033_MOESM2_ESM.pptx]

## Slide 1
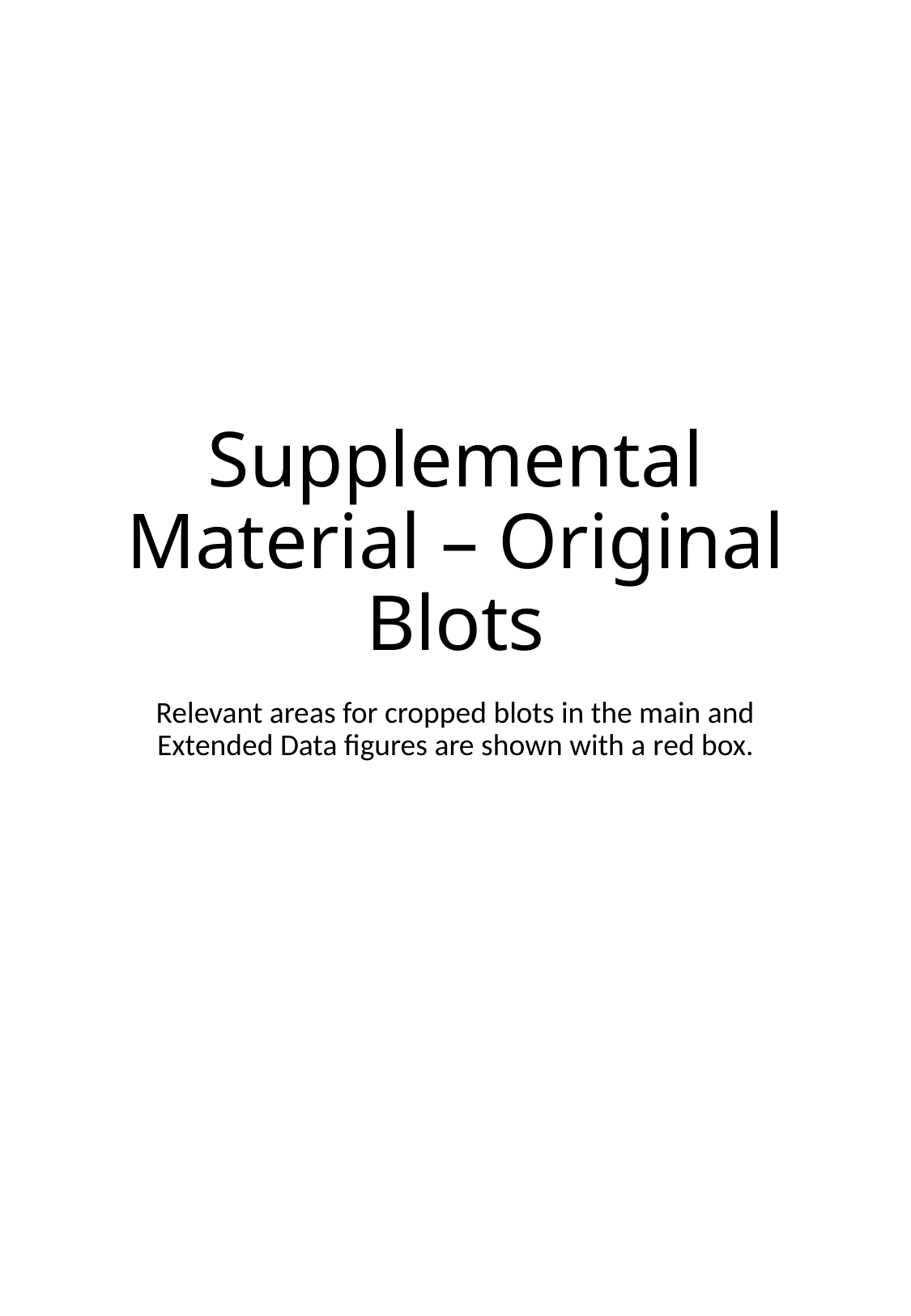

# Supplemental Material – Original Blots
Relevant areas for cropped blots in the main and Extended Data figures are shown with a red box.

## Slide 2
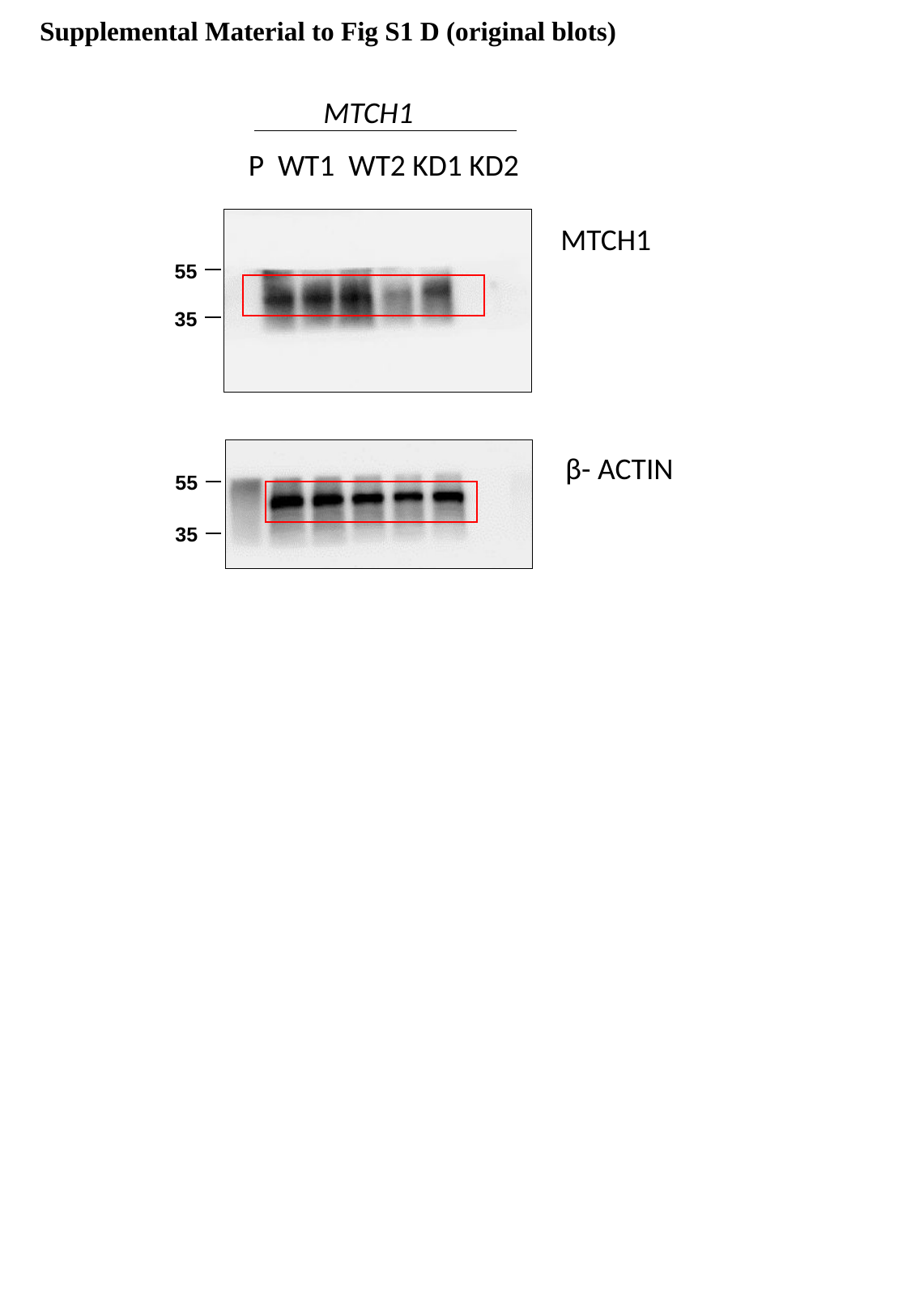

Supplemental Material to Fig S1 D (original blots)
MTCH1
P WT1 WT2 KD1 KD2
MTCH1
55
35
β- ACTIN
55
35

## Slide 3
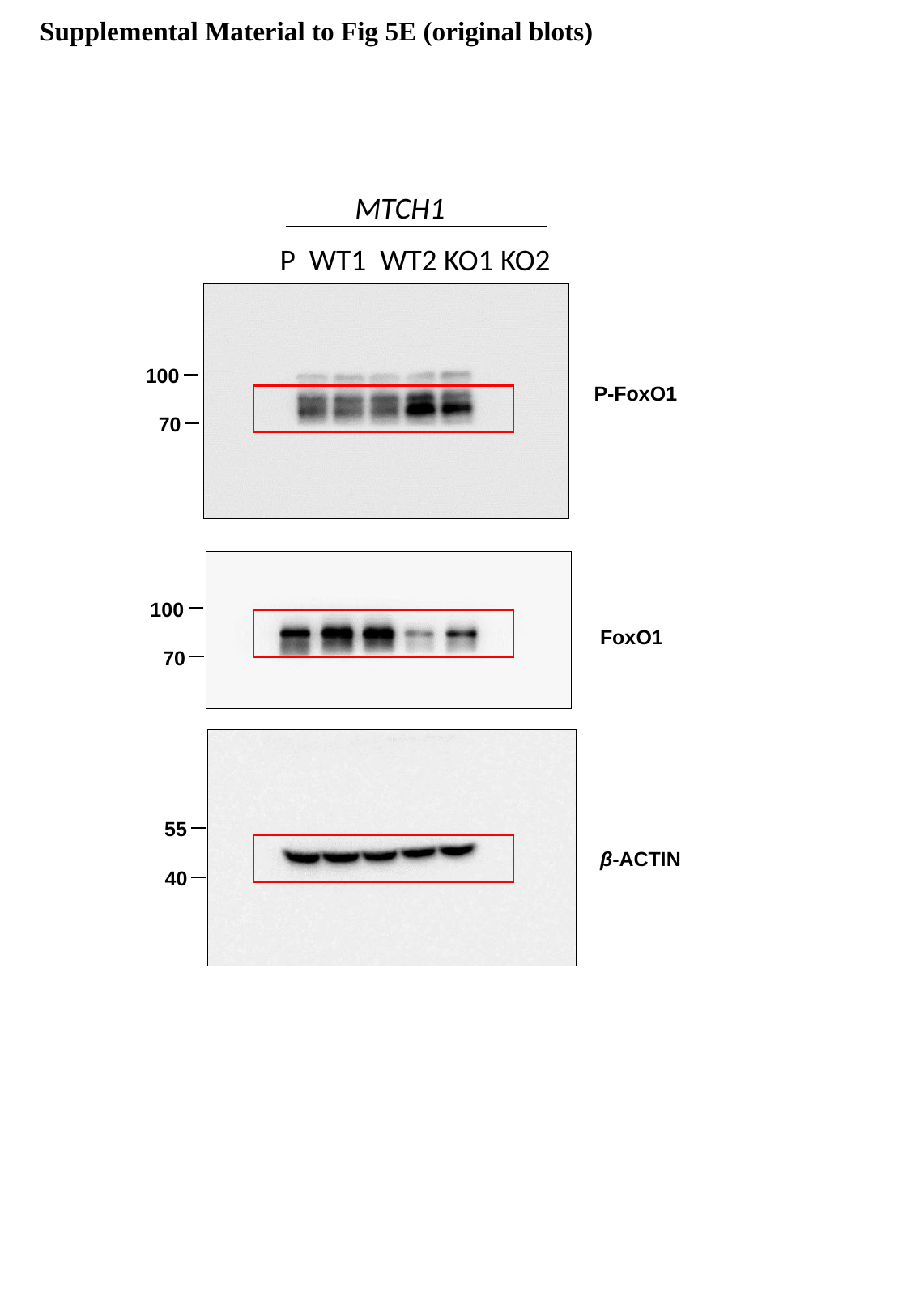

Supplemental Material to Fig 5E (original blots)
MTCH1
P WT1 WT2 KO1 KO2
100
P-FoxO1
70
100
FoxO1
70
55
β-ACTIN
40

## Slide 4
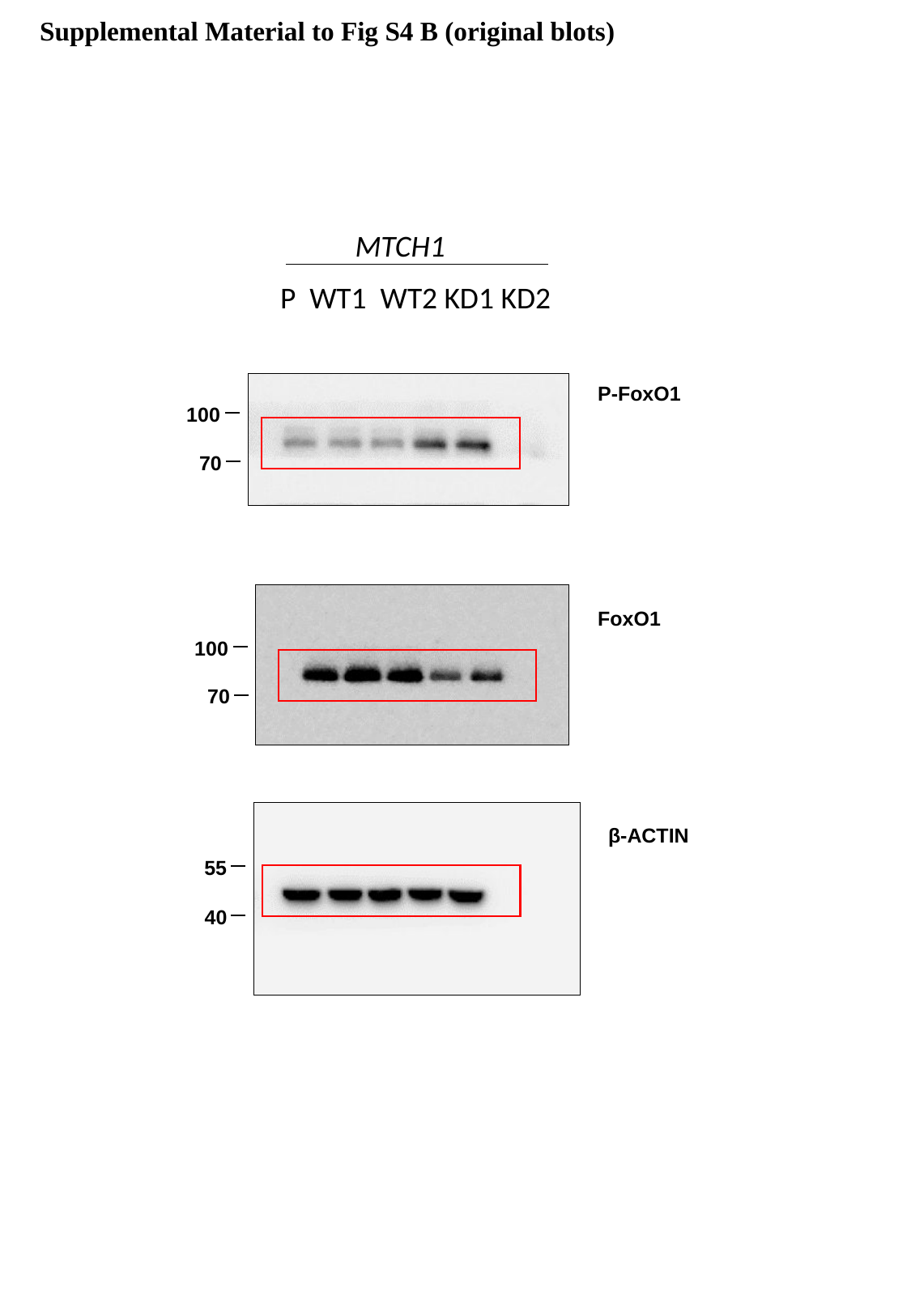

Supplemental Material to Fig S4 B (original blots)
MTCH1
P WT1 WT2 KD1 KD2
P-FoxO1
100
70
FoxO1
100
70
β-ACTIN
55
40

## Slide 5
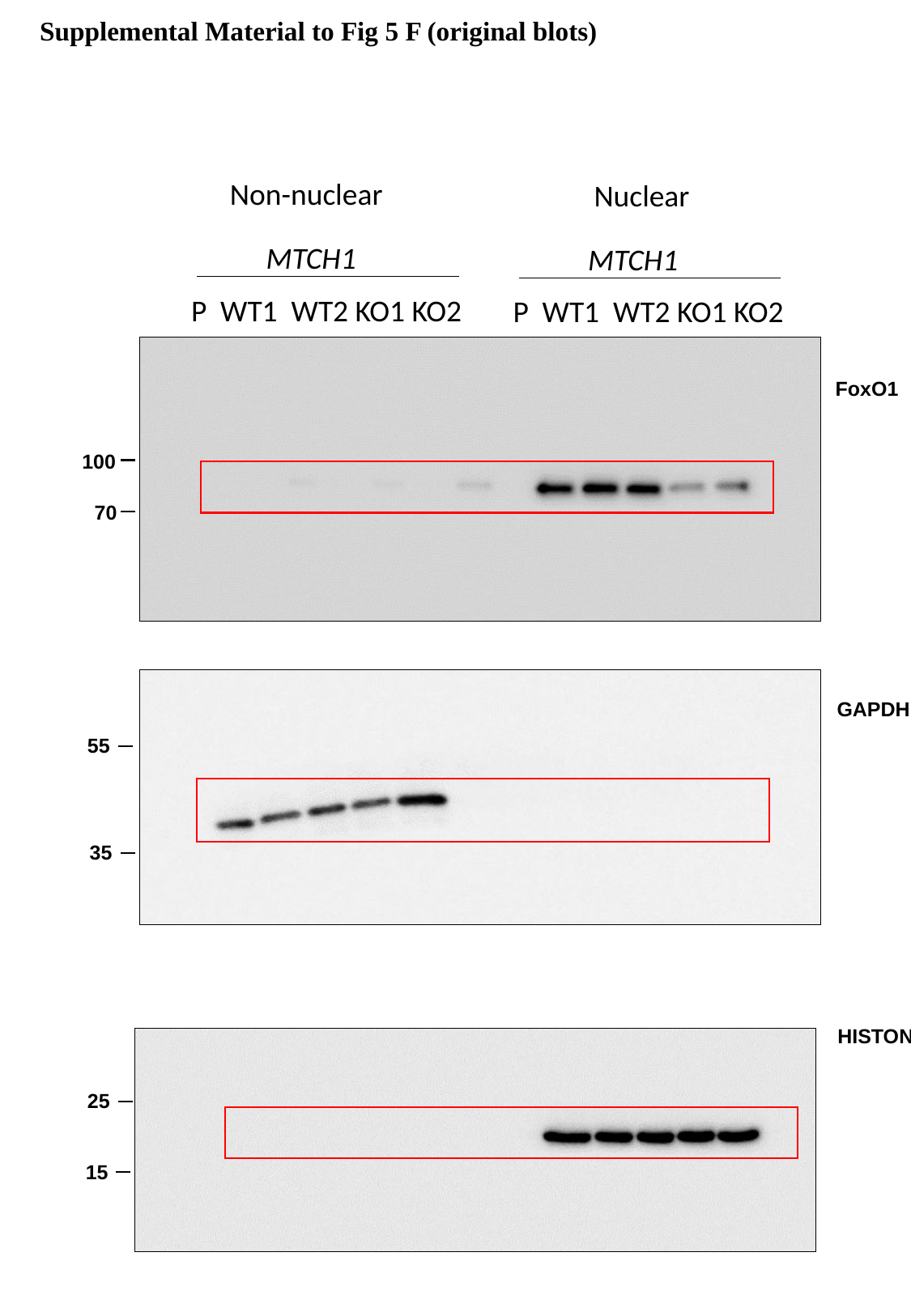

Supplemental Material to Fig 5 F (original blots)
Non-nuclear
Nuclear
MTCH1
P WT1 WT2 KO1 KO2
MTCH1
P WT1 WT2 KO1 KO2
FoxO1
100
70
GAPDH
55
35
HISTONE
25
15

## Slide 6
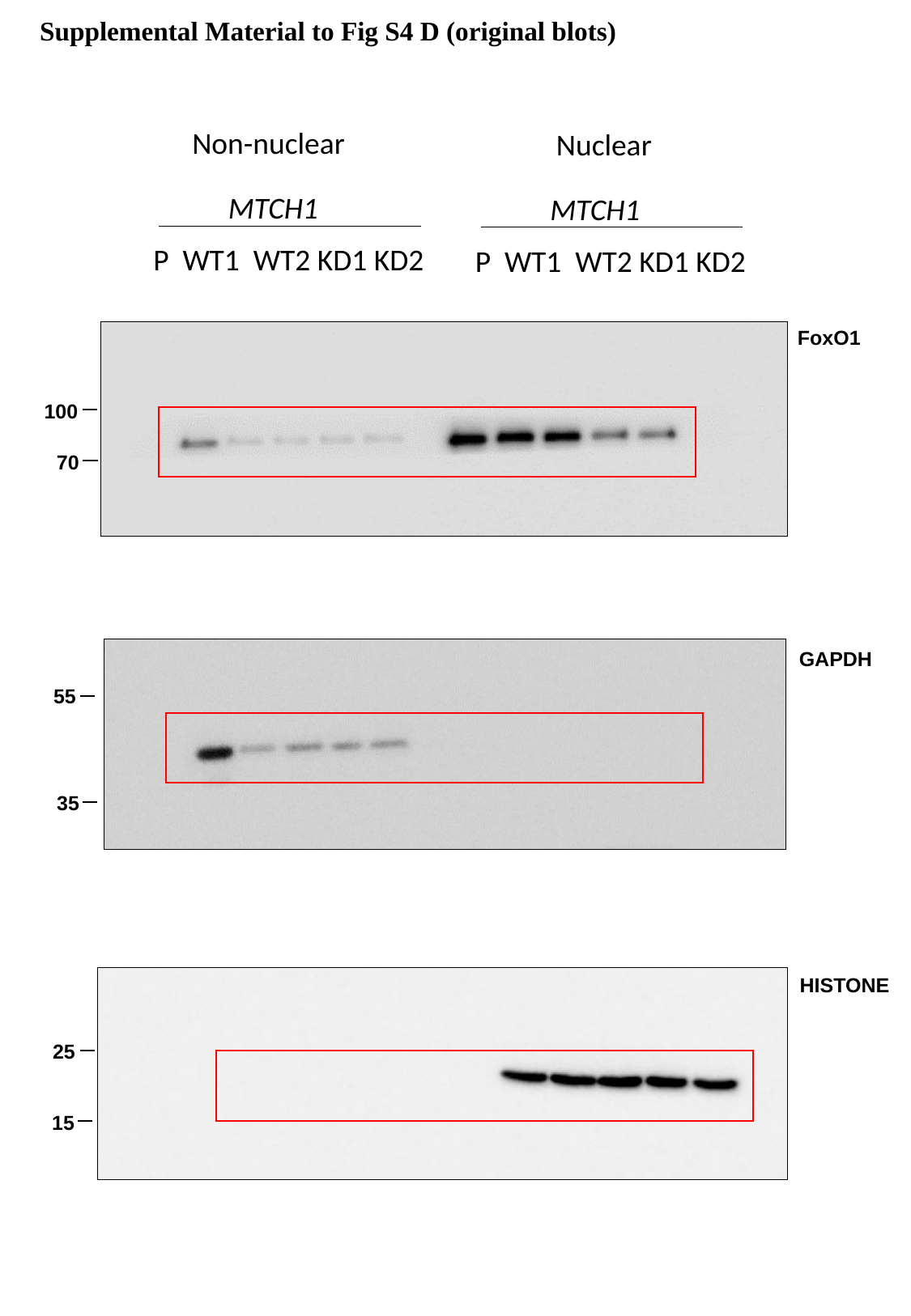

Supplemental Material to Fig S4 D (original blots)
Non-nuclear
Nuclear
MTCH1
P WT1 WT2 KD1 KD2
MTCH1
P WT1 WT2 KD1 KD2
FoxO1
100
70
GAPDH
55
35
HISTONE
25
15

## Slide 7
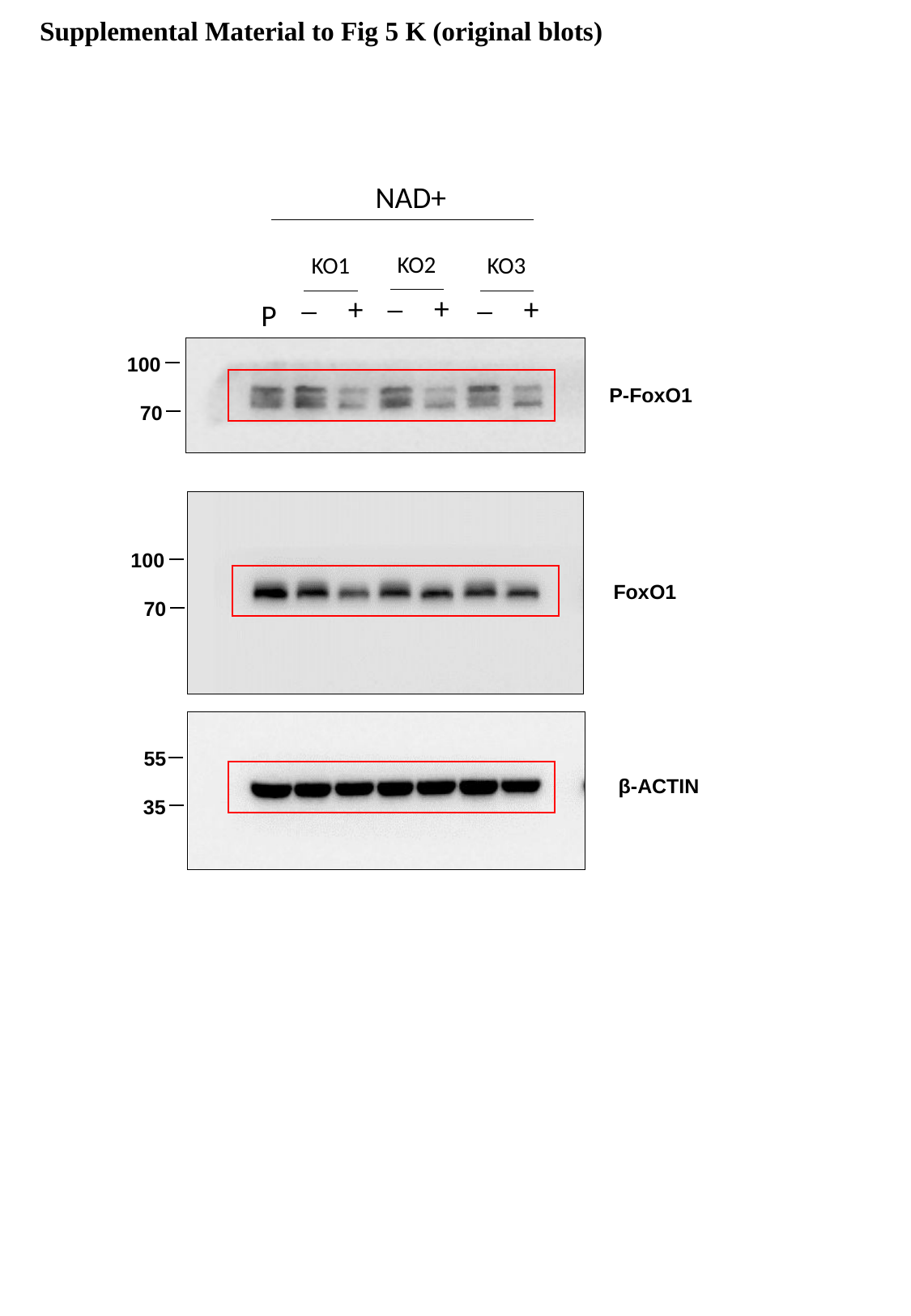

Supplemental Material to Fig 5 K (original blots)
NAD+
KO2
_
+
KO1
_
+
KO3
_
+
P
100
P-FoxO1
70
100
FoxO1
70
55
β-ACTIN
35

## Slide 8
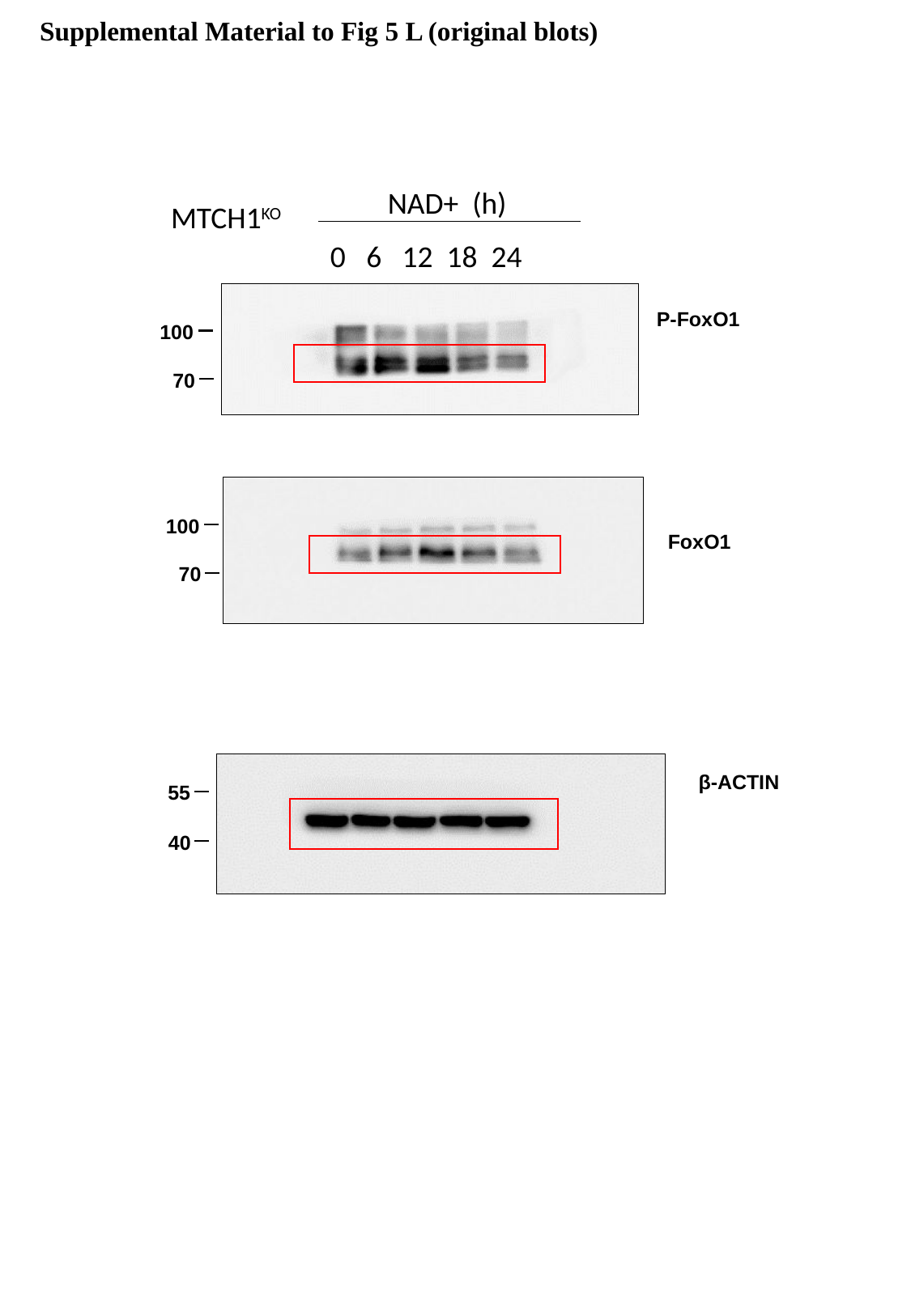

Supplemental Material to Fig 5 L (original blots)
NAD+ (h)
MTCH1KO
0 6 12 18 24
100
70
P-FoxO1
100
70
FoxO1
β-ACTIN
55
40

## Slide 9
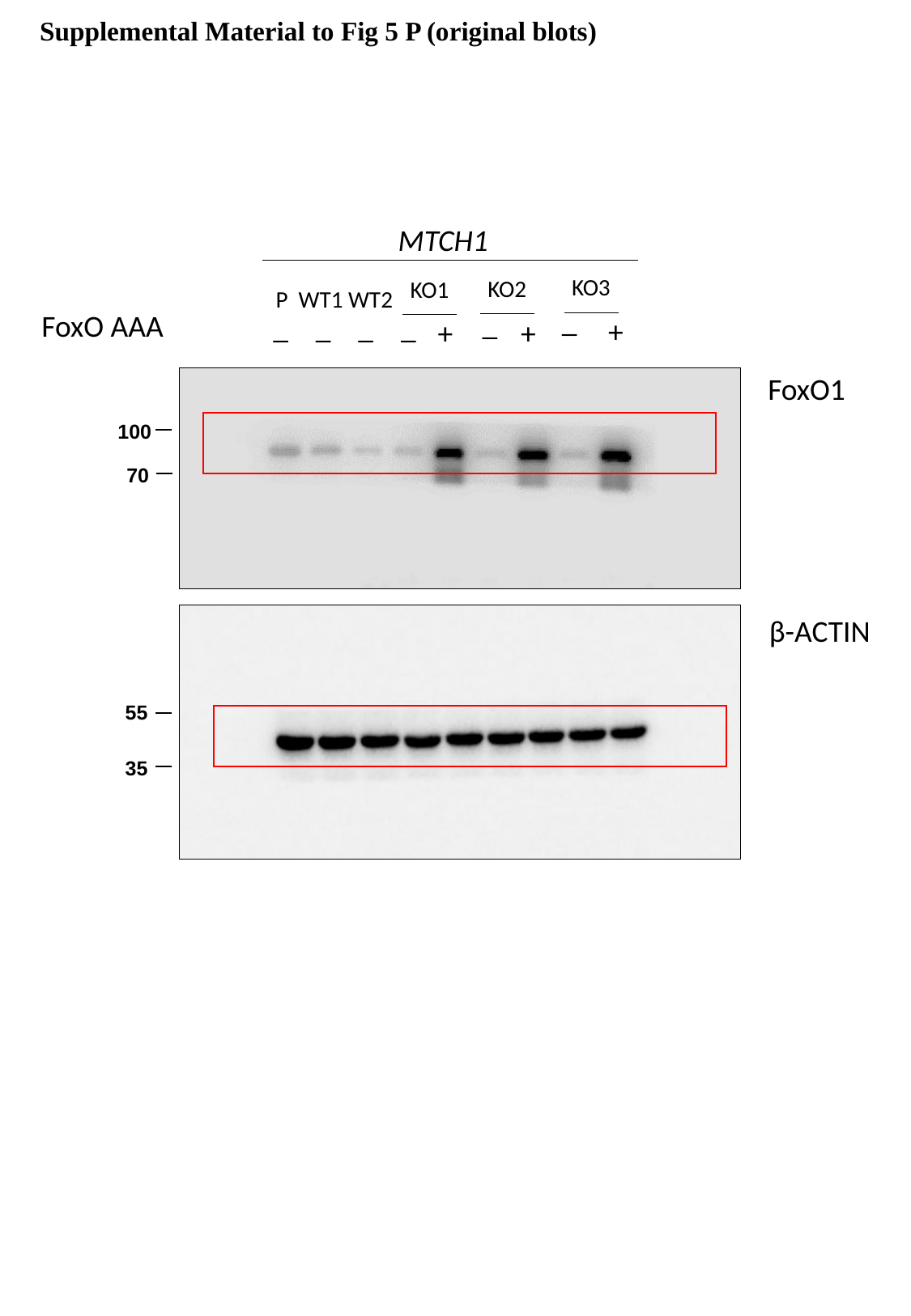

Supplemental Material to Fig 5 P (original blots)
MTCH1
KO3
_
+
KO2
KO1
P WT1 WT2
_
FoxO AAA
_ _ _ _
+
+
FoxO1
100
70
β-ACTIN
55
35
