# Supplementary material for: Mitochondrial carrier 1 (MTCH1) governs ferroptosis by triggering the FoxO1-GPX4 axis-mediated retrograde signaling in cervical cancer cells
Source: Cell Death Dis. 2023 Aug 8;14(8):508. doi: 10.1038/s41419-023-06033-2 (PMC10406804; doi:10.1038/s41419-023-06033-2)
Supplement: Supplementary file 4 — Author change confirmation [file 41419_2023_6033_MOESM4_ESM.pdf]

主　题:	Re: Author change request for the manuscript entitled"Mitochondrial carrier 1 (MTCH1) governs ferroptosis by triggering the FoxO1-GPX4 axis-mediated retrograde signaling in cervical cancer cells"
发件人:	"Wang Xuan" <xuanwxuan@outlook.com> 2023-8-1 10:04:36
收件人:	"Junjie Luo" <luojj@cau.edu.cn>

Dear Prof.Luo,
Thank you very much for the request.
I agree and confirm the author change.
Best regards
Xuan Wang

获取 Outlook for iOS

发件人: Junjie Luo <luojj@cau.edu.cn>
发送时间: Tuesday, August 1, 2023 9:41:45 AM
收件人: xuanwxuan@outlook.com <xuanwxuan@outlook.com>
主题: Author change request for the manuscript entitled"Mitochondrial carrier 1 (MTCH1) governs ferroptosis by triggering the FoxO1-GPX4 axis-mediated retrograde signaling in cervical cancer cells"

Dear Dr. Xuan Wang,

I hope this email finds you well. I am writing to request a change in the authorship for our manuscript entitled "Mitochondrial carrier 1 (MTCH1) governs ferroptosis by triggering the FoxO1-GPX4 axis-mediated retrograde signaling in cervical cancer cells", which was submitted to Cell Death & Disease. The manuscript ID is CDDIS-23-0803R.

We would like to add Zhenglong Gu as an author due to his valuable advice and guidance for manuscript improvement. The revised author list would be as follows:

Xuan Wang[1,#], Yuting Ji[1,2,3,#], Jingyi Qi[1], Shuaishuai Zhou[1], Sitong Wan[1], Chang Fan[1], Zhenglong Gu[2,3], Peng An[1,*], Yongting Luo[1,*] and Junjie Luo[1,*]

1.Department of Nutrition and Health, Beijing Advanced Innovation Center for Food Nutrition and Human Health, China Agricultural University, Beijing 100193, China

2. Greater Bay Area Institute of Precision Medicine (Guangzhou), Fudan University, Nansha District, Guangzhou 511400, China

3. Institute of Life Sciences, Fudan University, Shanghai 200433, China

* Correspondence: luojj@cau.edu.cn (J.L.); luo.yongting@cau.edu.cn (Y.L.); an-peng@cau.edu.cn (P.A.)

# These authors contributed equally to this work.

Please reply to this email to agree and confirm the author change.

Thank you for your time and support.

Sincerely,

Junjie Luo

主　题:	Re: Author change request for the manuscript entitled"Mitochondrial carrier 1 (MTCH1) governs ferroptosis by triggering the FoxO1-GPX4 axis-mediated retrograde signaling in cervical cancer cells"
发件人:	季玉婷 <sy20213313322@cau.edu.cn> 2023-8-1 14:39:48
收件人:	"Junjie Luo" <luojj@cau.edu.cn>

Dear Prof. Luo,

Thank you very much for the request. I agree and confirm the author change.

Best regards.

Yuting Ji

-----原始邮件-----
发件人: "Junjie Luo" <luojj@cau.edu.cn>
发送时间: 2023-08-01 09:42:47 (星期二)
收件人: sy20213313322@cau.edu.cn
主题: Author change request for the manuscript entitled "Mitochondrial carrier 1 (MTCH1) governs ferroptosis by triggering the FoxO1-GPX4 axis-mediated retrograde signaling in cervical cancer cells "

Dear Ms. Yuting Ji,

I hope this email finds you well. I am writing to request a change in the authorship for our manuscript entitled "Mitochondrial carrier 1 (MTCH1) governs ferroptosis by triggering the FoxO1-GPX4 axis-mediated retrograde signaling in cervical cancer cells", which was submitted to Cell Death & Disease. The manuscript ID is CDDIS-23-0803R.

We would like to add Zhenglong Gu as an author due to his valuable advice and guidance for manuscript improvement. The revised author list would be as follows:

Xuan Wang[1,#], Yuting Ji[1,2,3,#], Jingyi Qi[1], Shuaishuai Zhou[1], Sitong Wan[1], Chang Fan[1], Zhenglong Gu[2,3], Peng An[1,*], Yongting Luo[1,*] and Junjie Luo[1,*]

1.Department of Nutrition and Health, Beijing Advanced Innovation Center for Food Nutrition and Human Health, China Agricultural University, Beijing 100193, China

2. Greater Bay Area Institute of Precision Medicine (Guangzhou), Fudan University, Nansha District, Guangzhou 511400, China

3. Institute of Life Sciences, Fudan University, Shanghai 200433, China

* Correspondence: luojj@cau.edu.cn (J.L.); luo.yongting@cau.edu.cn (Y.L.); an-peng@cau.edu.cn (P.A.)

# These authors contributed equally to this work.

Please reply to this email to agree and confirm the author change.

Thank you for your time and support.

Sincerely,

Junjie Luo

主　题:	Re:Author change request for the manuscript entitled"Mitochondrial carrier 1 (MTCH1) governs ferroptosis by triggering the FoxO1-GPX4 axis-mediated retrograde signaling in cervical cancer cells"
发件人:	中国农业大学齐静宜 <qipeiyan2992@163.com> 2023-8-1 9:54:01
收件人:	"Junjie Luo" <luojj@cau.edu.cn>

Dear Prof. Luo,
Thank you very much for the request. I agree and confirm the author change.
Have a nice day!
Jingyi Qi

At 2023-08-01 09:43:26, "Junjie Luo" <luojj@cau.edu.cn> wrote:

> Dear Ms. Jingyi Qi,
>
> I hope this email finds you well. I am writing to request a change in the authorship for our manuscript entitled "Mitochondrial carrier 1 (MTCH1) governs ferroptosis by triggering the FoxO1-GPX4 axis-mediated retrograde signaling in cervical cancer cells", which was submitted to Cell Death & Disease. The manuscript ID is CDDIS-23-0803R.
>
> We would like to add Zhenglong Gu as an author due to his valuable advice and guidance for manuscript improvement. The revised author list would be as follows:
>
> Xuan Wang[1,#], Yuting Ji[1,2,3,#], Jingyi Qi[1], Shuaishuai Zhou[1], Sitong Wan[1], Chang Fan[1], Zhenglong Gu[2,3], Peng An[1,*], Yongting Luo[1,*] and Junjie Luo[1,*]
>
> 1.Department of Nutrition and Health, Beijing Advanced Innovation Center for Food Nutrition and Human Health, China Agricultural University, Beijing 100193, China
>
> 2. Greater Bay Area Institute of Precision Medicine (Guangzhou), Fudan University, Nansha District, Guangzhou 511400, China
>
> 3. Institute of Life Sciences, Fudan University, Shanghai 200433, China
>
> * Correspondence: luojj@cau.edu.cn (J.L.); luo.yongting@cau.edu.cn (Y.L.); an-peng@cau.edu.cn (P.A.)
>
> # These authors contributed equally to this work.
>
> **Please reply to this email to agree and confirm the author change.**
>
> Thank you for your time and support.
>
> Sincerely,
>
> Junjie Luo

主　题:	Re: Author change request for the manuscript entitled"Mitochondrial carrier 1 (MTCH1) governs ferroptosis by triggering the FoxO1-GPX4 axis-mediated retrograde signaling in cervical cancer cells"
发件人:	周帅帅 <shuaishuaizhou@cau.edu.cn> 2023-8-1 10:22:10
收件人:	"Junjie Luo" <luojj@cau.edu.cn>

Dear Prof. Luo,

Thank you very much for the request. At Zhenglong Gu's suggestions, the quality of manuscript has been improved. So, I agree and confirm the author change.

Best regards,

Shuaishuai Zhou

> -----原始邮件-----
> 发件人: "Junjie Luo" <luojj@cau.edu.cn>
> 发送时间: 2023-08-01 09:44:00 (星期二)
> 收件人: shuaishuaizhou@cau.edu.cn
> 主题: Author change request for the manuscript entitled "Mitochondrial carrier 1 (MTCH1) governs ferroptosis by triggering the FoxO1-GPX4 axis-mediated retrograde signaling in cervical cancer cells "
>
> Dear Ms. Shuaishuai Zhou,
>
> I hope this email finds you well. I am writing to request a change in the authorship for our manuscript entitled "Mitochondrial carrier 1 (MTCH1) governs ferroptosis by triggering the FoxO1-GPX4 axis-mediated retrograde signaling in cervical cancer cells", which was submitted to Cell Death & Disease. The manuscript ID is CDDIS-23-0803R.
>
> We would like to add Zhenglong Gu as an author due to his valuable advice and guidance for manuscript improvement. The revised author list would be as follows:
>
> Xuan Wang[1,#], Yuting Ji[1,2,3,#], Jingyi Qi[1], Shuaishuai Zhou[1], Sitong Wan[1], Chang Fan[1], Zhenglong Gu[2,3], Peng An[1,*], Yongting Luo[1,*] and Junjie Luo[1,*]
>
> 1.Department of Nutrition and Health, Beijing Advanced Innovation Center for Food Nutrition and Human Health, China Agricultural University, Beijing 100193, China
>
> 2. Greater Bay Area Institute of Precision Medicine (Guangzhou), Fudan University, Nansha District, Guangzhou 511400, China
>
> 3. Institute of Life Sciences, Fudan University, Shanghai 200433, China
>
> * Correspondence: luojj@cau.edu.cn (J.L.); luo.yongting@cau.edu.cn (Y.L.); an-peng@cau.edu.cn (P.A.)
>
> # These authors contributed equally to this work.
>
> **Please reply to this email to agree and confirm the author change.**
>
> Thank you for your time and support.
>
> Sincerely,
>
> Junjie Luo

主　题:	Re: Author change request for the manuscript entitled"Mitochondrial carrier 1 (MTCH1) governs ferroptosis by triggering the FoxO1-GPX4 axis-mediated retrograde signaling in cervical cancer cells"
发件人:	adeline7wan <adeline7wan@163.com> 2023-8-1 10:05:34
收件人:	"Junjie Luo" <luojj@cau.edu.cn>

Dear Prof. Luo,

Thank you very much for the request. I agree and confirm the author change.
Have a nice day!

Sitong Wan

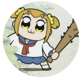

adeline7wan

邮箱：adeline7wan@163.com

---- Replied Message ----

From	Junjie Luo<luojj@cau.edu.cn>
Date	08/01/2023 09:44
To	adeline7wan<adeline7wan@163.com>
Subject	Author change request for the manuscript entitled"Mitochondrial carrier 1 (MTCH1) governs ferroptosis by triggering the FoxO1-GPX4 axis-mediated retrograde signaling in cervical cancer cells"

Dear Ms. Sitong Wan,

I hope this email finds you well. I am writing to request a change in the authorship for our manuscript entitled "Mitochondrial carrier 1 (MTCH1) governs ferroptosis by triggering the FoxO1-GPX4 axis-mediated retrograde signaling in cervical cancer cells", which was submitted to Cell Death & Disease. The manuscript ID is CDDIS-23-0803R.

We would like to add Zhenglong Gu as an author due to his valuable advice and guidance for manuscript improvement. The revised author list would be as follows:

Xuan Wang[1,#], Yuting Ji[1,2,3,#], Jingyi Qi[1], Shuaishuai Zhou[1], Sitong Wan[1], Chang Fan[1], Zhenglong Gu[2,3], Peng An[1,*], Yongting Luo[1,*] and Junjie Luo[1,*]

1.Department of Nutrition and Health, Beijing Advanced Innovation Center for Food Nutrition and Human Health, China Agricultural University, Beijing 100193, China

2. Greater Bay Area Institute of Precision Medicine (Guangzhou), Fudan University, Nansha District, Guangzhou 511400, China

3. Institute of Life Sciences, Fudan University, Shanghai 200433, China

* Correspondence: luojj@cau.edu.cn (J.L.); luo.yongting@cau.edu.cn (Y.L.); an-peng@cau.edu.cn (P.A.)

# These authors contributed equally to this work.

Please reply to this email to agree and confirm the author change.

Thank you for your time and support.

Sincerely,

Junjie Luo

主　题:	Re:Author change request for the manuscript entitled"Mitochondrial carrier 1 (MTCH1) governs ferroptosis by triggering the FoxO1-GPX4 axis-mediated retrograde signaling in cervical cancer cells"
发件人:	fanchang <18035829041@163.com> 2023-8-1 14:49:50
收件人:	"Junjie Luo" <luojj@cau.edu.cn>

Dear Prof. Luo

Thank you very much for the request.I agree and confirm the author change.

Best regards

Chang Fan

At 2023-08-01 09:45:09, "Junjie Luo" <luojj@cau.edu.cn> wrote:

> Dear Ms. Chang Fan,
>
> I hope this email finds you well. I am writing to request a change in the authorship for our manuscript entitled "Mitochondrial carrier 1 (MTCH1) governs ferroptosis by triggering the FoxO1-GPX4 axis-mediated retrograde signaling in cervical cancer cells", which was submitted to Cell Death & Disease. The manuscript ID is CDDIS-23-0803R.
>
> We would like to add Zhenglong Gu as an author due to his valuable advice and guidance for manuscript improvement. The revised author list would be as follows:
>
> Xuan Wang[1,#], Yuting Ji[1,2,3,#], Jingyi Qi[1], Shuaishuai Zhou[1], Sitong Wan[1], Chang Fan[1], Zhenglong Gu[2,3], Peng An[1,*], Yongting Luo[1,*] and Junjie Luo[1,*]
>
> 1.Department of Nutrition and Health, Beijing Advanced Innovation Center for Food Nutrition and Human Health, China Agricultural University, Beijing 100193, China
>
> 2. Greater Bay Area Institute of Precision Medicine (Guangzhou), Fudan University, Nansha District, Guangzhou 511400, China
>
> 3. Institute of Life Sciences, Fudan University, Shanghai 200433, China
>
> * Correspondence: luojj@cau.edu.cn (J.L.); luo.yongting@cau.edu.cn (Y.L.); an-peng@cau.edu.cn (P.A.)
>
> # These authors contributed equally to this work.
>
> **Please reply to this email to agree and confirm the author change.**
>
> Thank you for your time and support.
>
> Sincerely,
>
> Junjie Luo

主　题:	Re: Author change request for the manuscript entitled"Mitochondrial carrier 1 (MTCH1) governs ferroptosis by triggering the FoxO1-GPX4 axis-mediated retrograde signaling in cervical cancer cells"
发件人:	顾正龙 <guzhenglong@ipm-gba.org.cn> 2023-8-1 11:11:32
收件人:	"Junjie Luo" <luojj@cau.edu.cn>

Dear Junjie,

Thanks for your email. Yeah, I would love to coauthor this paper due to our previous discussion. Very good work,

Zhenglong

-----原始邮件-----
发件人: "Junjie Luo" <luojj@cau.edu.cn>
发送时间: 2023-08-01 09:40:17 (星期二)
收件人: guzhenglong@ipm-gba.org.cn
主题: Author change request for the manuscript entitled "Mitochondrial carrier 1 (MTCH1) governs ferroptosis by triggering the FoxO1-GPX4 axis-mediated retrograde signaling in cervical cancer cells "

Dear Professor Zhenglong Gu,

I hope this email finds you well. I am writing to request a change in the authorship for our manuscript entitled "Mitochondrial carrier 1 (MTCH1) governs ferroptosis by triggering the FoxO1-GPX4 axis-mediated retrograde signaling in cervical cancer cells", which was submitted to Cell Death & Disease. The manuscript ID is CDDIS-23-0803R.

We would like to add Zhenglong Gu as an author due to his valuable advice and guidance for manuscript improvement. The revised author list would be as follows:

Xuan Wang[1,#], Yuting Ji[1,2,3,#], Jingyi Qi[1], Shuaishuai Zhou[1], Sitong Wan[1], Chang Fan[1], Zhenglong Gu[2,3], Peng An[1,*], Yongting Luo[1,*] and Junjie Luo[1,*]

1.Department of Nutrition and Health, Beijing Advanced Innovation Center for Food Nutrition and Human Health, China Agricultural University, Beijing 100193, China

2. Greater Bay Area Institute of Precision Medicine (Guangzhou), Fudan University, Nansha District, Guangzhou 511400, China

3. Institute of Life Sciences, Fudan University, Shanghai 200433, China

* Correspondence: luojj@cau.edu.cn (J.L.); luo.yongting@cau.edu.cn (Y.L.); an-peng@cau.edu.cn (P.A.)

# These authors contributed equally to this work.

Please reply to this email to agree and confirm the author change.

Thank you for your time and support.

Sincerely,

Junjie Luo

主　题:	Re: Author change request for the manuscript entitled"Mitochondrial carrier 1 (MTCH1) governs ferroptosis by triggering the FoxO1-GPX4 axis-mediated retrograde signaling in cervical cancer cells"
发件人:	"Peng An" <an-peng@cau.edu.cn> 2023-8-1 10:14:25
收件人:	"Junjie Luo" <luojj@cau.edu.cn>

Dear Junjie,

I agree and confirm the author change.

Thanks!

Peng

在 2023年8月1日，09:37，Junjie Luo <luojj@cau.edu.cn> 写道:

Dear Professor Peng An,

I hope this email finds you well. I am writing to request a change in the authorship for our manuscript entitled "Mitochondrial carrier 1 (MTCH1) governs ferroptosis by triggering the FoxO1-GPX4 axis-mediated retrograde signaling in cervical cancer cells", which was submitted to Cell Death & Disease. The manuscript ID is CDDIS-23-0803R.

We would like to add Zhenglong Gu as an author due to his valuable advice and guidance for manuscript improvement. The revised author list would be as follows:

Xuan Wang[1,#], Yuting Ji[1,2,3,#], Jingyi Qi[1], Shuaishuai Zhou[1], Sitong Wan[1], Chang Fan[1], Zhenglong Gu[2,3], Peng An[1,*], Yongting Luo[1,*] and Junjie Luo[1,*]

1.Department of Nutrition and Health, Beijing Advanced Innovation Center for Food Nutrition and Human Health, China Agricultural University, Beijing 100193, China

2. Greater Bay Area Institute of Precision Medicine (Guangzhou), Fudan University, Nansha District, Guangzhou 511400, China

3. Institute of Life Sciences, Fudan University, Shanghai 200433, China

* Correspondence: luojj@cau.edu.cn (J.L.); luo.yongting@cau.edu.cn (Y.L.); an-peng@cau.edu.cn (P.A.)

# These authors contributed equally to this work.

Please reply to this email to agree and confirm the author change.

Thank you for your time and support.

Sincerely,

Junjie Luo

主　题:	Re: Author change request for the manuscript entitled"Mitochondrial carrier 1 (MTCH1) governs ferroptosis by triggering the FoxO1-GPX4 axis-mediated retrograde signaling in cervical cancer cells"
发件人:	罗永挺 <luo.yongting@cau.edu.cn> 2023-8-1 9:38:15
收件人:	"Junjie Luo" <luojj@cau.edu.cn>

Dear Prof. Luo,

Thank you very much for the request.

I agree and confirm the author change.

Best regards

Yongting Luo

-----原始邮件-----
发件人: "Junjie Luo" <luojj@cau.edu.cn>
发送时间: 2023-08-01 09:26:01 (星期二)
收件人: luo.yongting@cau.edu.cn
主题: Author change request for the manuscript entitled "Mitochondrial carrier 1 (MTCH1) governs ferroptosis by triggering the FoxO1-GPX4 axis-mediated retrograde signaling in cervical cancer cells "

Dear Professor Yongting Luo,

I hope this email finds you well. I am writing to request a change in the authorship for our manuscript entitled "Mitochondrial carrier 1 (MTCH1) governs ferroptosis by triggering the FoxO1-GPX4 axis-mediated retrograde signaling in cervical cancer cells", which was submitted to Cell Death & Disease. The manuscript ID is CDDIS-23-0803R.

We would like to add Zhenglong Gu as an author due to his valuable advice and guidance for manuscript improvement. The revised author list would be as follows:

Xuan Wang[1,#], Yuting Ji[1,2,3,#], Jingyi Qi[1], Shuaishuai Zhou[1], Sitong Wan[1], Chang Fan[1], Zhenglong Gu[2,3], Peng An[1,*], Yongting Luo[1,*] and Junjie Luo[1,*]

1.Department of Nutrition and Health, Beijing Advanced Innovation Center for Food Nutrition and Human Health, China Agricultural University, Beijing 100193, China

2. Greater Bay Area Institute of Precision Medicine (Guangzhou), Fudan University, Nansha District, Guangzhou 511400, China

3. Institute of Life Sciences, Fudan University, Shanghai 200433, China

* Correspondence: luojj@cau.edu.cn (J.L.); luo.yongting@cau.edu.cn (Y.L.); an-peng@cau.edu.cn (P.A.)

# These authors contributed equally to this work.

Please reply to this email to agree and confirm the author change.

Thank you for your time and support.

Sincerely,

Junjie Luo

主　题:	Re: Author change request for the manuscript entitled"Mitochondrial carrier 1 (MTCH1) governs ferroptosis by triggering the FoxO1-GPX4 axis-mediated retrograde signaling in cervical cancer cells"
发件人:	"Junjie Luo" <luojj@cau.edu.cn> 2023-8-1 10:01:43
收件人:	luojj@cau.edu.cn

Dear Prof. Luo,

Thanks for your letter. I agree and confirm the author change.

Best regards

Junjie Luo

-----原始邮件-----
发件人: "Junjie Luo" <luojj@cau.edu.cn>
发送时间: 2023-08-01 09:39:09 (星期二)
收件人: luojj@cau.edu.cn
主题: Author change request for the manuscript entitled "Mitochondrial carrier 1 (MTCH1) governs ferroptosis by triggering the FoxO1-GPX4 axis-mediated retrograde signaling in cervical cancer cells "

Dear Professor Junjie Luo,

I hope this email finds you well. I am writing to request a change in the authorship for our manuscript entitled "Mitochondrial carrier 1 (MTCH1) governs ferroptosis by triggering the FoxO1-GPX4 axis-mediated retrograde signaling in cervical cancer cells", which was submitted to Cell Death & Disease. The manuscript ID is CDDIS-23-0803R.

We would like to add Zhenglong Gu as an author due to his valuable advice and guidance for manuscript improvement. The revised author list would be as follows:

Xuan Wang[1,#], Yuting Ji[1,2,3,#], Jingyi Qi[1], Shuaishuai Zhou[1], Sitong Wan[1], Chang Fan[1], Zhenglong Gu[2,3], Peng An[1,*], Yongting Luo[1,*] and Junjie Luo[1,*]

1.Department of Nutrition and Health, Beijing Advanced Innovation Center for Food Nutrition and Human Health, China Agricultural University, Beijing 100193, China

2. Greater Bay Area Institute of Precision Medicine (Guangzhou), Fudan University, Nansha District, Guangzhou 511400, China

3. Institute of Life Sciences, Fudan University, Shanghai 200433, China

* Correspondence: luojj@cau.edu.cn (J.L.); luo.yongting@cau.edu.cn (Y.L.); an-peng@cau.edu.cn (P.A.)

# These authors contributed equally to this work.

Please reply to this email to agree and confirm the author change.

Thank you for your time and support.

Sincerely,

Junjie Luo
